# Metal–Organic Frameworks and Their Composites for Environmental Applications

**DOI:** 10.1002/advs.202204141

**Published:** 2022-09-14

**Authors:** Qian Zhang, Hui Yang, Ting Zhou, Xudong Chen, Wenting Li, Huan Pang

**Affiliations:** ^1^ School of Chemistry and Chemical Engineering Yangzhou University Yangzhou 225009 China

**Keywords:** air purification, environment, metal–organic frameworks, water treatment

## Abstract

From the point of view of the ecological environment, contaminants such as heavy metal ions or toxic gases have caused harmful impacts on the environment and human health, and overcoming these adverse effects remains a serious and important task. Very recent, highly crystalline porous metal–organic frameworks (MOFs), with tailorable chemistry and excellent chemical stability, have shown promising properties in the field of removing various hazardous pollutants. This review concentrates on the recent progress of MOFs and MOF‐based materials and their exploit in environmental applications, mainly including water treatment and gas storage and separation. Finally, challenges and trends of MOFs and MOF‐based materials for future developments are discussed and explored.

## Graphical abstract

This review focuses on the recent progress of metal–organic frameworks (MOFs) and MOF‐based materials and their exploit in environmental applications, mainly including water treatment and gas storage and separation. In addition, challenges and trends of MOFs and MOF‐based materials for future developments are discussed and explored.

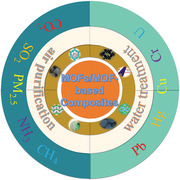

## 1 Introduction

The high speed growth of population worldwide and extensive consumption of fossil fuel have caused climate change and the serious pollution of the environment.^[^ ^1^, ^2^, ^3^, ^4^, ^5^ ^]^ The overuse of chemical fertilizers, which caused the generation of residual and hazardous substances^[^ ^6^ ^]^ and the emission of toxic gases, threatens human health.^[^ ^7^ ^]^ The illness and diseases resulted from the environment contamination have threatened the sustainable development of human society.^[^ ^8^ ^]^ Consequently, multiple technologies for advanced water and air cleaning have been developed to purify the environment and satisfy the increasing energy demand.^[^ ^9^ ^]^

In recent years, various precious metal‐based electrocatalysts were utilized for water decomposition, however, the characteristics of the precious metals finally restrict their usage.^[^ ^10^ ^]^ Carbon fixation was employed to reduce CO_2_ emissions and diminish greenhouse effect, yet this method is costly and not conducive to large‐scale production.^[^ ^11^ ^]^ Electrochemistry strategy was used to reduce CO_2_ to hydrocarbons, whereas, it is a complicated multistep process involving adsorbed intermediates and great kinetic obstacles.^[^ ^12^ ^]^ Hydropower, sunlight and wind energy are also carried out to replace fossil fuel energy for generating energy. Nevertheless, these energy cannot be collected or released while in need, and they are greatly affected by the weather. In addition, dwindling freshwater pollution from industrial wastewater has also been one of the most critical global environmental issues, especially heavy metal pollution.^[^ ^13^, ^14^ ^]^ In light of the situations mentioned above, it is extremely urgent to develop efficient and durable materials for practical applications in environmental purification.

In the past decade, a new class of crystalline porous metal–organic frameworks (MOFs), have been studied in widespread applications such as adsorbent,^[^ ^15^, ^16^, ^17^, ^18^, ^19^, ^20^ ^]^ photoreduction catalyst,^[^ ^21^, ^22^, ^23^, ^24^ ^]^ rapid removal kinetics,^[^ ^25^ ^]^ catalyst,^[^ ^26^, ^27^, ^28^ ^]^ scavenger,^[^ ^29^ ^]^ and separation substance and environmental purifications,^[^ ^17^, ^30^, ^31^, ^32^, ^33^, ^34^, ^35^ ^]^ because of their adjustable inner surface, tailorable morphology,^[^ ^36^ ^]^ hydrolysis stability,^[^ ^37^ ^]^ adjustable hole/cage size, large specific surface area,^[^ ^38^ ^]^ and flexible skeleton.^[^ ^39^ ^]^ Compared with traditional inorganic porous materials (activated carbon, zeolite, etc.), MOFs have the merits of wider photocatalytic absorption range and effective controllable preparation of microporous structure.^[^ ^40^, ^41^ ^]^ MOFs are crystal compounds composed of organic linkers and infinite lattice or secondary building units (SBUs, metal ions or clusters), which are connected by coordination bonds with moderate strength.^[^ ^42^, ^43^, ^44^, ^45^, ^46^, ^47^, ^48^, ^49^ ^]^ In order to improve the performance of MOFs, all kinds of types of functional materials including polymers, carbons, and graphene, have been integrated with MOFs to generate MOF‐based composites. Due to synergistic effects, the resulting MOF composites combine the intrinsic characteristic of each component and exhibit novel physicochemical properties.

**Scheme 1:**
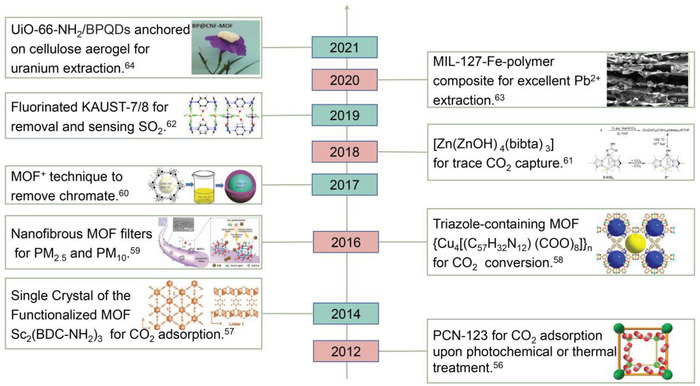
Timeline of MOFs and MOF‐based materials for environment applications.

In this review, we summarize the most recent progresses toward the synthesis of MOFs and MOF‐based materials and their applications in environment purification,^[^ ^50^, ^51^, ^52^, ^53^, ^54^, ^55^ ^]^ especially in water and air environment (**Scheme** **1**).^[^ ^56^, ^57^, ^58^, ^59^, ^60^, ^61^, ^62^, ^63^, ^64^ ^]^ The challenges and prospects of MOFs and MOF‐based materials for future practical application were critically discussed. Compared with the related reviews in this field, this review particularly emphasize the preparation strategies and the application of MOFs and MOF‐based composites in the environmental field.^[^ ^65^, ^66^, ^67^ ^]^ We expect that this review will benefit readers to rationally design and develop MOFs and MOF‐based materials for environmental applications.

## 2 Synthetic Strategies of MOFs and MOF‐Based Materials

MOFs, or more generally porous coordination polymers, have been well known as a novel type of porous materials, but it was not until 1990s that this field received great attention, especially because of the pioneering work of Kitagawa and co‐workers,^[^ ^68^, ^69^ ^]^ Yaghi and co‐workers,^[^ ^70^, ^71^ ^]^ and Férey, who first determined the full potential of this ordered solid family. It is a remarkable fact that MOFs have unique porous structure, ordered porosity, rapid mass transfer efficiency,^[^ ^72^ ^]^ high mechanical strength,^[^ ^73^, ^74^ ^]^ good antibacterial characteristic,^[^ ^75^ ^]^ strong degradation ability,^[^ ^76^, ^77^ ^]^ excellent adsorption performance,^[^ ^78^, ^79^ ^]^ and strong catalytic performance.^[^ ^79^, ^80^ ^]^ Due to these superior properties,^[^ ^81^ ^]^ MOFs have become an ideal and potential material for a variety of sustainable technologies and applications in the last decade, such as chemical sensing, drug transportation, catalysis, gas separation and storage, and water treatment.^[^ ^82^ ^]^ However, the commercialization of MOFs and most of its potential applications, primarily depends on the availability of the synthesis and manufacturing procedures over the consideration of sustainability and environmental impact.^[^ ^83^ ^]^ So far, more than 20 000 original MOFs structures have been reported, some of which have been produced by BASF on a tonnage scale.^[^ ^82^ ^]^

Importantly, the composite material has great potentialities for environmental applications. Below, we will emphatically focus on several synthetic strategies of MOFs and MOF‐based materials.

### 2.1 Hydrothermal Method

Hydrothermal synthetic method is common to synthesize inorganic materials.^[^ ^84^, ^85^ ^]^ Solvents are selected to make raw materials into solutions, which are packaged in hydrothermal kettle and heated to 100–200 °C. The materials synthesized under hydrothermal conditions exhibit excellent properties.^[^ ^86^, ^87^ ^]^ During the reaction process, the hydrothermal kettle in the system keeps a certain self‐generated pressure, and the water phase reaction in nonequilibrium hydrothermal system can usually produce porous nanomaterials.^[^ ^88^ ^]^ Hydrothermal method is less corrosive to equipment, and the amount of downstream fermentation inhibitor is low.^[^ ^89^ ^]^

**Figure 1:**
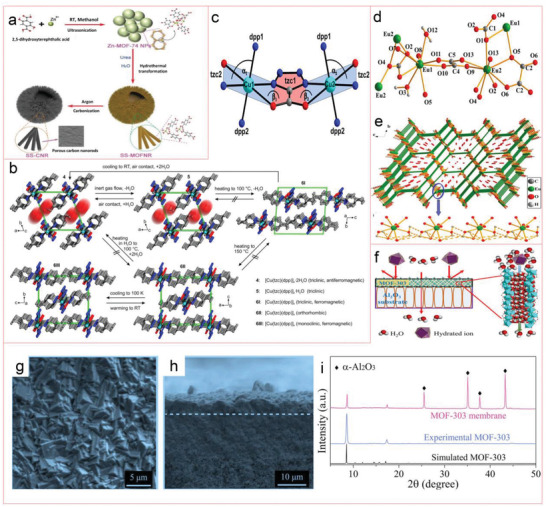
a) Schematic figure of the formation of SS‐MOFNRSS‐CNR. Reproduced with permission.^[^ ^90^ ^]^ Copyright 2019, Wiley‐VCH. b) Crystal construction of [Cu(tzc)(dpp)]*_n_* ·2H_2_O, [Cu(tzc)(dpp)]*_n_* ·H_2_O and its polymorphous anhydrate modifications [Cu(tzc)(dpp)]*_n_*. c) The Cu(II) coordination environment of the compounds. Reproduced with permission.^[^ ^91^ ^]^ Copyright 2014, American Chemical Society. d) Characterization of Eu^3+^ coordination environment in crystal structure. e) The crystal structure for [Eu_2_(CO_3_)(ox)_2_(H_2_O)_2_]·4H_2_O showing the 1D channels along the *a*‐axis filled with the free waters; the ox groups are simplified as the green bonds. Reproduced with permission.^[^ ^92^ ^]^ Copyright 2014, American Chemical Society. f) Schematic diagram of water desalination with an MOF‐303 membrane. g,h) Top view and cross‐sectional SEM images of an MOF‐303 membrane. i) XRD patterns of experimental MOF‐303 powder and membrane. Reproduced with permission.^[^ ^93^ ^]^ Copyright 2021, American Chemical Society.

In a recent study, crystalline Zn‐MOF‐74 nanoparticles with an average diameter of 30–50 nm were prepared by ultrasonication‐assisted method as precursors and urea as regulators to produce chestnut shell‐like spherical superstructure of MOF nanorods (SS‐MOFNR) in water through hydrothermal transformation. The SS‐MOFNR was carbonized in argon flow, and the original form was kept, providing spherical superstructure of carbon nanorods (SS‐CNRs) with 1D porous carbon nanorods assembled into 3D spherical superstructure (**Figure** **1**a).^[^ ^90^ ^]^ Wu et al. dissolved Ni(NO_3_)_2_·4H_2_O and 1‐H‐benzimidazole‐5‐carboxylic acid (HBIC) in a H_2_O/CH_3_CN mixture to prepare Ni(HBIC)_2_·2.5H_2_O (JUC‐86) (JUC = Jilin University) with 3D structure and high thermal stability.^[^ ^94^ ^]^ In addition, Hall et al. suspended the dried powder in a mixture of 10 mL ethanol and water (volume ratio 1:1), put it into a stainless steel autoclave lined with 23 mL polytetrafluoroethylene, and heated it at 200 °C for 20 h. After hydrothermal treatment, a series of operations, such as filtering the sample, were carried out to obtain a microporous titanium dioxide replica. Without hydrothermal treatment, only amorphous titanium dioxide was obtained.^[^ ^95^ ^]^ By hydrothermal treatment of Cu(tzc) with dpp (tzc = tetrazolate‐5‐carboxylate, dpp = 1,3‐di(4‐pyridyl)propane) in a single reaction flask, heterogeneous mixtures of three different single crystal solvates [Cu(tzc)(dpp)]*_n_* ·0.5C_6_H_14_·0.5H_2_O, [Cu(tzc)(dpp)]*_n_* ·4.5H_2_O, and [Cu(tzc)(dpp)]*_n_* ·1.25C_6_H_14_ (Figure 1b) were obtained. In their crystalline texture, each Cu(II) cation passes through two symmetrically connected tzc coordination ligands and two octahedral geometrically symmetric dpp ligands, while the adjacent Cu(II) cations are spined by one tzc and one dpp ligand, respectively, forming a 1D chain (Figure 1c).^[^ ^91^ ^]^ As shown in Figure 1d,e, a lanthanide MOF compound [Eu_2_(Co_3_)(ox)_2_(H_2_O)_2_]·4H_2_O in good yield was prepared by hydrothermal method. The Eu(1) atom is nonacoordinate, coordinated with five oxalate, two carbonate, and two hydrated O atoms (O3 and O12); Eu1–O distances range from 2.430(5) to 2.654(4), among which two distances relating to water ligands ((Eu1–O3 = 2.546(3) Å and Eu1–O12 = 2.550(3) Å) are significantly longer than those containing oxalic acid ligand and excluding Eu1–O5 (2.654). Each carbonyl ligand bridges one Eu(1) atom and three Eu(2) atoms, which are combined in a form of *µ*_4_–*η* ^2^:*η* ^2^:*η*,^2^ forming a double‐sided sawtooth lanthanide carbonate chain structure.^[^ ^92^ ^]^ By in situ hydrothermal method, MIL‐101(Cr)@MCM‐41 composite was successfully obtained with enhanced gas adsorption performance and good reusability.^[^ ^96^ ^]^

Very recently, continuous MOF‐303 membrane supported on porous alumina disk was synthesized by in situ hydrothermal synthesis method. The as‐prepared membrane shows high divalent ion rejection rate and unprecedented permeability, especially with good stability in brine. It can be used for water desalination and alleviate the lack of fresh water (Figure 1f–i), and its crystal structure and morphology are well preserved. The MOF‐303 composite is recommended as an excellent film material for water softening.^[^ ^93^ ^]^

### 2.2 Solvothermal Method

The principle of solvothermal method resembles that of hydrothermal method, but the difference is that the solvent used is not limited to water.^[^ ^97^, ^98^, ^99^, ^100^ ^]^ There are three main steps: 1) uniformly dispersing the raw materials in an organic solvents; 2) transferring the mixture into a sealed autoclave and setting the reaction temperature; and 3) cooling, solvent washing, centrifuging, and finally drying to obtain the final material.^[^ ^88^ ^]^ MOF‐based materials synthesized by solvothermal method has higher specific surface area and larger pore volume.^[^ ^101^, ^105^ ^]^ However, the solvothermal process is not only time‐consuming and expensive, but also difficult to control.^[^ ^102^, ^106^ ^]^

**Figure 2:**
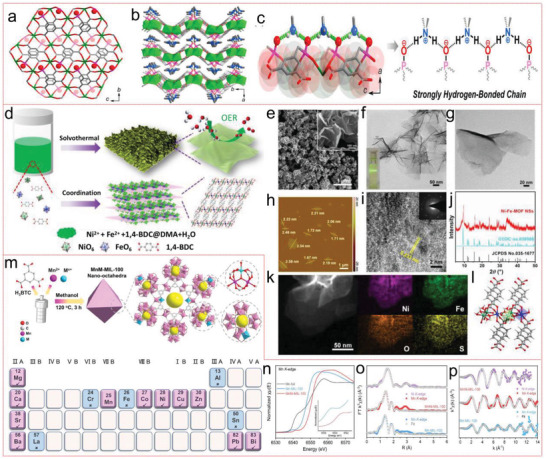
a) A perspective view of (Me_2_NH_2_)[Eu(L)] along the *a*‐axis (the uncoordinated O atoms of phosphonate groups are shown as red spheres) and b) sandwich structure along the *c*‐axis, with counter‐cations (Me_2_NH_2_) periodically arranged in the interlayer. c) A strong hydrogen bond (N—H···O) chain composed of (Me_2_NH_2_)^+^ cations of phosphonate groups in anionic host framework and uncoordinated oxygen (O7) atoms alternately. Reproduced with permission.^[^ ^103^ ^]^ Copyright 2017, American Chemical Society. d) Synthetic procedure for ultrathin MOF their utilization and nanosheets for the oxygen evolution reaction. e) SEM image. f,g) TEM images. h) AFM image. i) HRTEM image. j) PXRD pattern. k) EDX elemental mappings and HAADF‐STEM image and l) crystal structure of the Ni‐Fe‐MOF NSs. Reproduced with permission.^[^ ^107^ ^]^ Copyright 2019, Wiley‐VCH. m) Schematic figure of the ordinary synthetic process for MnM‐MIL‐100. n) Normalized XANES spectra of Mn‐MIL‐100 and MnNi‐MIL‐100 at the Mn K‐edge. The corresponding EXAFS data o) *R*‐space and p) *k*‐space fitting curves. Reproduced with permission.^[^ ^108^ ^]^ Copyright 2021, Wiley‐VCH.

Wei et al. reported a proton‐conducting MOF, (Me_2_NH_2_)[Eu(L)] (H_4_L = 5‐(phosphonomethyl)isophthalic acid) prepared via solvothermal method, which manifests remarkable anhydrous conductivity (**Figure** **2**a–c).^[^ ^103^ ^]^ Most importantly, proton‐transfer mechanism in MOFs and other conductive materials is directly observed and well‐established by both anisotropic conductivity test and control experiments. In another precedent, highly stable MOF composites composed of 12‐tungstophoric acid (HPW) and Zr‐benzene tricarboxylate (HPW@Zr‐BTC) were successfully prepared by a one‐pot solvothermal method. The acylation reaction of benzoyl chloranisole was catalyzed by composite materials. In the solvent‐free condition, 28.2 wt% HPW@Zr‐BTC has excellent catalytic function, and the conversion rate of anisole can reach 99.4% and the yield can reach 97.6% (p‐methoxybenzophenone). Due to the unobservable HPW leaching, the catalytic activity of the catalyst was well preserved after at least five consecutive runs.^[^ ^104^ ^]^

Recently, Li et al. synthesized Ni‐M‐MOF (M = Fe, Al, Co, Mn, Zn, and Cd) nanosheets (NSs) with a few atomic layers in thickness by bottom‐up solvothermal method at a large‐scale. The addition of mixed solvents of DMF and water were important to the controllable synthesis of these MOF NSs, which further employed as an effective electrocatalyst with high performance (Figure 2d–l).^[^ ^107^ ^]^ More recently, a series of Mn‐based multimetallic MOF (bimetallic and trimetallic MIL‐100) nano‐octahedrons were successfully synthesized by a one‐pot solvothermal strategy, with uniform sizes for highly efficient energy storage (Figure 2m–p).^[^ ^108^ ^]^ ZnIn_2_S_4_@NH_2_‐MIL‐125(Ti) prepared through solvothermal method has high photocatalytic activity.^[^ ^109^ ^]^ Su et al. prepared nanocomposite Cd_0.2_Zn_0.8_S@UiO‐66‐NH_2_ with different UiO‐66‐NH_2_ content by simple solvothermal method. The composite has good photocatalytic activity for hydrogen evolution and CO_2_ reduction.^[^ ^110^ ^]^

### 2.3 The Microwave/Ultrasound‐Assisted Synthesis Method

Microwave/ultrasonic‐assisted synthesis method is considered as a friendly and efficient strategy. This method can not only ensure the yield of MOFs products, but also shorten the crystallization time and reaction time, so it has been widely utilized.^[^ ^102^ ^]^ The expected MOF‐based material can be produced by the following ways: the mixed raw material source containing organic ligands and metal ions are reacted under the condition of ultrasonic or microwave with certain power.^[^ ^88^ ^]^

Three MOFs, IRMOF‐1, 2, and 3 assembled from Zn(NO_3_)_2_·6H_2_O and 1,4‐benzenedicarboxylic acid (BDCH_2_) were reported via microwave‐assisted processes. This strategy can not only shorten the reaction time but also increase the yield from ≈30% to over 90%. The microwave technique facilitated the nucleation process leading to a narrow size distribution due to that all crystals are nucleated immediately.^[^ ^111^ ^]^ Similarly, a lanthanide 2D MOF, [Dy(MeCOO)(PhCOO)_2_]*_n_* nanosheets, was synthesized by a simple microwave‐assisted synthesis method based on the principle that neutral nanosheets are heaped into microcrystals by van der Waals force interaction. The material demonstrates single‐ion‐magnet behavior, while its magnetically diluted analogue [La_0.9_Dy_0.1_(MeCOO)(PhCOO)_2_] is a multifunctional, luminescent, and magnetic 2D compound. By sonication, [Dy(MeCOO)(PhCOO)_2_]*_n_* nanosheets can be exfoliated into stable lamellae.^[^ ^112^ ^]^

### 2.4 Room Temperature Conversion Method

The room temperature conversion method is relatively general and simple.^[^ ^113^, ^114^, ^116^ ^]^ Typically, Osterrieth et al. successfully prepared AuNR@Zr‐MOF composites by Zr‐MOF and AuNR through at room temperature conversion method. AuNR@MOFs can absorb or block molecules from pores, thus contributing to high selectivity sensing at the end of AuNR.^[^ ^115^ ^]^ In another example, bimetallic CoZn‐ZIF‐L with a leaf‐like structure was prepared at room temperature.^[^ ^117^ ^]^ CoFe(II)‐PBA‐HTPA was successfully prepared after a mild ligand exchange reaction between presynthesized ZIF‐67 TPAs and K_4_[Fe(CN)_6_] in aqueous solution at room temperature. The room temperature conversion process not only determined the formation of internal voids in nanostructures, but also provided doping of Fe atoms to CoP lattice, and CoFe(II)‐PBA‐HTPA has abundant active reaction sites.^[^ ^118^ ^]^

Recently, He et al. used room temperature conversion method to control electrochemical conversion of EC‐MOF film into porous and amorphous metal sulfide (a‐MS*_x_*).^[^ ^113^ ^]^ Riccò et al. obtained HKUST‐1 with excellent surface area in water/ethanol solution of copper carbonate and H_3_BTC at room temperature within 3 h.^[^ ^119^ ^]^ Hu et al. successfully prepared Co–N–C with tetragonal microstructures by adjusting the ratio of Co(CH_3_COO)_2_·4H_2_O and g‐C_3_N_4_ reactants and using simple solid‐state reaction principle and room temperature conversion method.^[^ ^277^ ^]^

**Figure 3:**
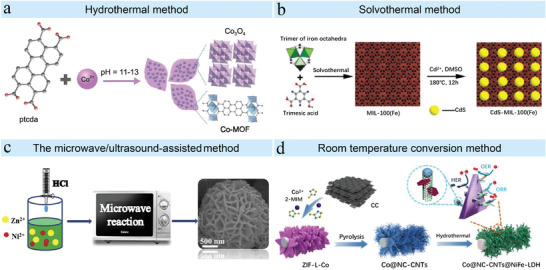
The typical synthetic strategies for MOFs and MOF composites. a) Hydrothermal method. Reproduced with permission.^[^ ^87^ ^]^ Copyright 2021, Oxford University Press. b) Solvothermal method. Reproduced with permission.^[^ ^106^ ^]^ Copyright 2015, Tsinghua University Press. c) The microwave/ultrasound‐assisted method. Reproduced with permission.^[^ ^102^ ^]^ Copyright 2018, Elsevier. d) Room temperature conversion method. Reproduced with permission.^[^ ^116^ ^]^ Copyright 2022, Elsevier.

In addition to the aforementioned common methods (**Figure** **3**), there are other techniques also that were used to synthesize MOFs and MOF‐based materials. Recently, Geng et al. have successfully prepared MIL‐96‐Al with kinds of shapes such as hexagonal lamellar crystal, hexagonal bicameral crystal, and hexagonal prism bicameral crystal by cosolvent method.^[^ ^120^ ^]^

## 3 Application of MOFs in Water Environmental Field

Moderate development of freshwater resources is essential for food security and human survival. But by some estimates, freshwater use is approaching its maximum edible value (the earth's freshwater boundary). Massive consumption of water makes it more difficult by the increasing population pressures, the vagaries of water use behavior and climate change.^[^ ^121^, ^122^, ^123^, ^124^ ^]^ Therefore, it is particularly imperative to control water pollution. Nowadays, MOFs have received huge attention as adsorbents for heavy and toxic metal ions with a small size because of their tailorable surface functionalities and high specific surface area.^[^ ^125^ ^]^ In the following sections, we sum up and expound different kinds of MOF‐based materials for efficient elimination of heavy metal ions (**Table** **1**).

**Table 1:** The adsorption capacity of different adsorbents for metal ions by MOF‐based materials

MOF‐based materials	Adsorption capacity [mg g^−1^]	Conditions	Reusability	Mechanism	Refs.
Lead ions					
CMC‐MOF/cloth	862.44	pH = 5	–	Complexation	[178]
PAN/chitosan/UiO‐66‐NH_2_	441.2	pH = 6	5	Complexation	[179]
Zn‐MOF	1097	pH = 4	6	Complexation	[135]
Ni_0.6_Fe_2.4_O_4_‐UiO‐66‐PEI	273.2	pH = 4	5	Electrostatic interaction/complexation	[180]
Fe_3_O_4_@TMU‐32	1600	C_0_ = 500 mg L^−1^, *v* = 25 mL, *m* = 5 mg, pH = 7	3	Complexation	[134]
BDB‐MIL‐125(Ti)@Fe_3_O_4_	710.79	pH = 6, *t* = 120 min	4	Electrostatic interaction/complexation	[130]
Mercury ions					
Zn(hip)(L)·(DMF)(H_2_O)	278	C_0_ = 100 mg L^−1^, *v* = 40 mL, m = 2 mg, *t* = 1 h	–	Chemisorption	[181]
LMOF‐263	380	–	–	Complexation	[182]
FJI‐H12	439.8	*m*(HgCl_2_) = 100 mg L^−1^, *v* = 50 mL, *m*_ads_ = 100 mg, *t* = 12 h	–	Complexation	[183]
MIL‐101‐Thymine	51.27	pH = 6	4	Complexation	[184]
Zr‐DMBD	171.5	pH = 6, *t* = 10 min	6	Proton exchange	[185]
In_2_S_3_@MIL‐101	518.2	C_0_ = 1000 mg L^−1^	3	Pore adsorption/complexation	[186]
Cys‐UiO‐66	350.14	*v* = 40 mL, *m* = 40 mg, pH = 5	5	Complexation	[187]
UiO‐66‐DMTD	670.5	C_0_ = 500 mg L^−1^, pH = 3	10	Complexation	[188]
Zr‐MSA	734	In a wide pH, *t* = 5 min	5	Complexation	[189]
Fe_3_O_4_@TMU‐32	905	C_0_ = 600 mg L^−1^, *v* = 25 mL, *m* = 5 mg, pH = 7	3	Complexation	[134]
Fe_3_O_4_@DTIM‐MOF@SH	756.9	*v* = 10 mL, *m* = 10 mg, *t* = 120 min	–	Complexation	[190]
TLMSM	954.7	In a wide pH, *m*/*V* = 0.5 g L^−1^	25	Complexation	[139]
Copper ions					
NH_2_‐MIL‐101(Al)@ZIF‐8	526.74	In a wide pH, C_0_ = 300 mg L^−1^	–	Complexation	[191]
ED‐MIL‐101(Cr)	69.9	C_0_ = 200 mg L^−1^, pH = 5.2 ± 0.2	3	Complexation	[143]
[(Zn_3_L_3_(H_2_O)_6_][(Na)(NO_3_)]	379.13	C_0_ = 10 µg mL^−1^, *v* = 80 mL, *m* = 10 mg pH = 6		Complexation	[142]
MOF‐199@PANI, core@shell	7831.34	C_0_ = 100 mg L^−1^, pH = 6, *T* = 20 °C		Complexation	[144]
Chromium ions					
ZJU‐101	118 [Cr(_VI_)]	–	–	Complexation	[192]
	245 (Cr_2_O_7_^2−^)	–	–	Complexation	
Ag_8_(tz)_6_](NO_3_)_2_·6H_2_O	37 [Cr(_VI_)]	pH = 6	–	Anion exchange	[193]
GO‐CS@[Zn(BDC)]	144.92 [Cr(_VI_)]	pH = 3	–	Electrostatic attraction	[194]
UiO‐66‐NH_2_@silica	277.4 (Cr_2_O_7_^2−^)	pH = 5	–	–	[195]
	133.4 [Cr(_VI_)]				
Zr‐BDC‐(NH_2_)_2_@PB	208 [Cr(_VI_)]	*t* = 35 min	5	Ion exchange	[147]
	432 (Cr_2_O_7_^2−^)	*t* = 35 min	5		
Uranyl ions					
GO‐COOH/UiO‐66	188.3	pH = 8, C_0_ = 95 mg L^−1^, *m*/*V* = 0.5 g L^−1^	5	Complexation; ion exchange	[196]
Fe_3_O_4_@ZIF‐8	523.5	pH = 3	–	Complexation	[197]
DSHM–DAMN	601	C_0_ = 400 mg L^−1^, pH = 8 *m*/*V* = 0.2	5	Complexation	[154]
GZA	602.4	pH = 7	–	Complexation/electrostatic interaction	[198]
AOPAN/ZIF	193.1	pH = 4, C_0_ = 100 mg L^−1^, *m*/*V* = 2 g L^−1^	–	Complexation	[162]
PN‐PCN‐222	756.1	*m*/*V* = 0.5 g L^−1^	–	Complexation/photocatalytic reduction	[153]
MIL‐101‐AO	586	pH = 7, C_0_ = 100 mg L^−1^	5	Complexation	
ZIF‐67/SAP	657.89			Complexation	[199]
MSONs‐5	526.6	pH = 6	5	Complexation	[149]
BP@CNFMOF	329.1	pH = 7		Complexation/photocatalytic reduction	[200]
UiO‐66‐3C4N	380.3	Uranium spiked simulated water (16 ppm)	–	Complexation/photocatalytic reduction	[161]
UiO‐66‐NH_2_@CS‐PDA	341.8	Uranium spiked simulated water (8 ppm)	–		
UiO‐66‐(COOH)4‐180	142.7	pH = 4, *m*/*V* = 1 g L^−1^	–	Complexation	[201]
ZIF‐90‐OM	610	pH = 5	5	Complexation	[202]
MOF‐808	80	pH = 1	3	Complexation; ion exchange	[203]
NU‐1000	110				
CMPM	5.81	Circulated seawater	6	Complexation	[204]
CaNDI‐*o*OH	572	pH = 4	>4	Complexation	[205]
MUU_im_	461	pH = 6	5	Complexation	[206]

### 3.1 Removal of Pb from Water

Excessive lead in drinking water is detrimental to human body, which will damage human organs and lead to permanent cognitive decline, slow action, and even brain injury. Lead ion, Pb(II) as Lewis acid, has no lone pair electrons and can receive electrons from Lewis base. Because of the tailorable surface functionalities, many researchers have studied superior MOFs and MOF‐based materials for removal of Pb(II).

**Figure 4:**
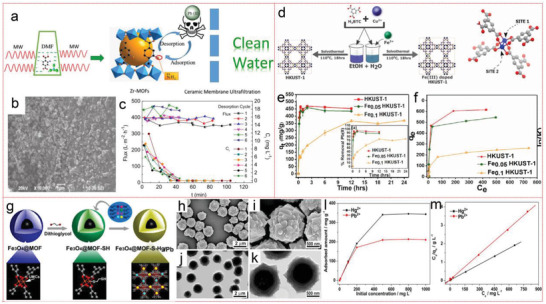
a) Schematic diagram of Zr‐MOFs‐CUF for effective removal of Pb(II). b) SEM images of the Zr–MOFs. c) Desorption cycles of the MOFs combined with the CUF membrane. Reproduced with permission.^[^ ^126^ ^]^ Copyright 2016, Elsevier. d) Schematic for illustrating doping in HKUST‐1 MOF, e) kinetics study, and f) isotherm study. Reproduced with permission.^[^ ^128^ ^]^ Copyright 2022, Elsevier. g) Schematic for the synthesis of thiol‐functionalized Fe_3_O_4_@Cu_3_(btc)_2_ microspheres and its application, h,i) SEM images and j,k) TEM images of Fe_3_O_4_@Cu_3_(btc)_2_, l) adsorption curves of Pb^2+^ and Hg^2+^ at different concentrations, and m) the Langmuir adsorption model. Reproduced with permission.^[^ ^127^ ^]^ Copyright 2017, Elsevier.

For example, amino‐functionalized Zr‐MOFs combining ceramic membrane ultrafiltration (CUF) was synthesized quickly (only 3 min) by microwave‐assisted method and the obtained MOFs molybdenum polysulfide is spherical with an average diameter of about 100 nm. Under the conditions of *T* = 35 °C, TMP = 0.15 MPa, and CFV = 4.0 m s^−1^, the removal rate of Pb(II) is the highest, reaching 61.4% with the lowest flux decline and membrane damage. The adsorption mechanism can be accounted for the coordination interaction between Pb(II) and amino group (—NH_2_). Controlling pH at 4.5 can effectively desorb Pb and cycle for six times. After removal, it can be cleaned with 0.5% ammonium citrate and 0.5% nitric acid solution at 18–50 °C for 1 h, and the final flux recovery efficiency is close to 100%, which possess superdurability (**Figure** **4**a–c).^[^ ^126^ ^]^

In addition, Ke et al. reported thiol‐functionalized magnetic core–shell Fe_3_O_4_@Cu_3_(btc)_2_ microspheres utilizing postsynthetic strategy, which show remarkable adsorption selectivity toward Pb(II) in the existence of other interfering ions and is potential to applied for the environmental pollution cleanup (Figure 4g–m).^[^ ^127^ ^]^ Furthermore, Goyal et al. explored the research of doping Fe into HKUST‐1 prepared by solvothermal method. Varying dopant concentrations, the partial substituted Fe‐HKUST‐1 shows higher surface area with low crystallinity. Thus Fe‐HKUST‐1 shows better Pb(II) selectivity than pristine HKUST‐1, removal rate up to 90% (Figure 4d–f).^[^ ^128^ ^]^

To develop a robust and high‐loading MOF functionalized nonwoven fabric composite, Zhao et al. successfully prepared UiO‐66‐NH_2_ in situ grown on the polyethylene terephthalate (PET) nonwoven fabric modified by polyacrylamide (PAM). The UiO‐66‐NH_2_‐PAM‐PET mixture shows an excellent adsorption capacity of 711.99 mg g^−1^ toward Pb(II), profiting by the intrinsic characteristic of UiO‐66‐NH_2_. The adsorption process was proved to monolayer adsorption and chemisorption.^[^ ^129^ ^]^ Recently, a novel BDB‐MIL‐125(Ti)@Fe_3_O_4_ by functionalized MIL‐125(Ti) with amino and thiol groups was designed. Benefiting from the abundant active sites, the maximum adsorption uptake was 710.79 mg g^−1^ at pH = 6 within 120 min with a maximum removal rate of 95.68%. Density functional theory (DFT) and the frontier molecular orbitals (FMOs) method manifests that the thiol group had a strong interaction for Pb(II) and facilitated to the charge transfer. The adsorption mechanism was mainly chelating and electrostation.^[^ ^130^ ^]^

### 3.2 Removal of Hg from Water

Usually, mercury plays a toxic role in human body in the form of divalent mercury ions. The harm of mercury is serious to human body and its poisoning can lead to acute symptoms, kidney damage, skin damage, and so on. Consequently, it is especially important to remove mercury from water.^[^ ^131^, ^132^ ^]^ Below, we introduce several examples using MOFs and MOF‐based materials to remove mercury from water.

**Figure 5:**
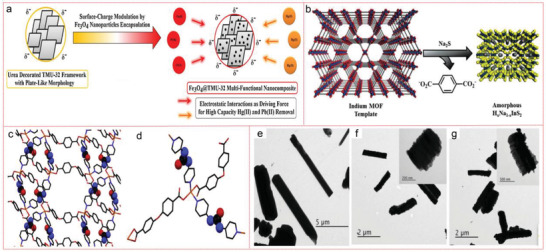
a) Proposed mechanism for Hg(II) and Pb(II) removal by Fe_3_O_4_ @TMU 32 composite. Structural representation of TMU‐32. b) TMU‐32 framework. c) Urea decorated motifs of TMU‐32. Reproduced with permission.^[^ ^134^ ^]^ Copyright 2020, Elsevier. d) Depiction of the wet‐treatment process to generate indium sulfide materials from sacrificial MOFs. Indium MOF templates MIL‐68 or MIL‐53‐NH_2_ are treated in aqueous or methanolic solutions of Na_2_S, which displaces the BDC ligand and results in formation of amorphous NaInS_2_ materials which retain the bulk morphology of the original MOF template. TEM images of MIL‐68: e) before treatment, and b,c) after treatment with Na_2_S in water f) or methanol g). The inset plots in (f) and (g) are magnified images of representative particles. Reproduced with permission.^[^ ^140^ ^]^ Copyright 2014, Wiley‐VCH.

For example, Ke et al. selected a well‐known 3D copper‐based MOF, namely, [Cu_3_(BTC)_2_(H_2_O)_3_]*_n_* (HKUST‐1, BTC = benzene‐1,3,5‐tricarboxylic acid salt), and carried out a simple thiol‐functionalized MOF based on the postcoordination synthesis. The thiol‐functionalized HKUST‐1 shows ultrahigh affinity (distribution efficiency, K_d_ = 4.73 ×10^5^ mL g^−1^) toward Hg(II) with high uptake of 714.29 mg g^−1^.^[^ ^133^ ^]^ To improve the recyclability and practicability, magnetic strategy by encapsulating Fe_3_O_4_ nanoparticles in TMU‐32 was carried out during in situ synthesis. Fe_3_O_4_@TMU‐32 nanocomposites show high capacity for Hg ions. This increase in capacity may be connected with the electrostatic interaction between the cationic properties of Hg and the negative charge of Fe_3_O_4_@TMU‐32 adsorbent. Because Fe_3_O_4_@TMU‐32 nanocomposites contain uniformly distributed charge modulation surfaces, it has strong reusability in the process of removing Hg for 3 cycles (**Figure** **5**a–c).^[^ ^134^ ^]^ Furthermore, Huang's group prepared an excellent MOF adsorbent with zinc ion and 3‐amino‐5‐mercapto‐1,2,4‐triazole with a very large surface area. The maximum absorption capacity of Zn‐MOF to Hg is 32 mg g^−1^ at pH = 5. The adsorption process of toxic metal ions by Zn‐MOF is completed by single‐layer adsorption of valence electron exchange on a uniform surface. Zn‐MOF can be recycled at least six times with high durability.^[^ ^135^ ^]^ Singh et al. functionalized MIL‐88A with mercapto ethanol to prepare a new MOF‐based adsorbent, MIL88A‐SH, to remove Hg(0) in air and Hg(II) in water. As an adsorbent, its adsorption capacity for Hg is very high, which can reach ≈1111.1 mg g^−1^. Its adsorption reaction kinetic is rapidly fast, and 95.5% of Hg is removed within 15 min. XRD, FTIR, and XPS studies show that Hg(0) is in situ oxidized to Hg(II) ions, and the interaction between Hg(II) and sulfhydryl groups facilitates the adsorption process. MIL88A‐SH has high adsorption capacity, excellent mercury removal, outstanding selectivity and recyclability, which is why it has become an effective adsorbent to solve Hg pollution.^[^ ^136^ ^]^ Similarly, Huang et al. realized the preparation of magnetic MOF composites (MFCs) with flexible mercapto suspension in the pores by solvent‐assisted ligand exchange, and the terephthalic acid in UIO‐66 was replaced by mercapto acetic acid, MFC‐S. The MFC‐S obtained shows a higher adsorption uptake (282 mg g^−1^) for Hg(II) ions than magnetic Zr‐MOF. MFC‐S is easy to be regenerated by liquid desorption after adsorbing metal ions, and can be reused for more than five times, with no obvious degradation in removal performance. Its durability is very high.^[^ ^137^ ^]^ At the same time, Mon et al. also use the hexagons modified by methionine residues as a strong and water‐stable MOF, which can reduce the concentration of Hg(II) ions in drinking water from a highly dangerous 10 mg L^−1^ to 27 µg L^−1^.^[^ ^138^ ^]^ Very recently, a thiol‐laced MOF‐based sponge monolith (TLMSM) with remarkable mechanical stability and chemical resistance, was developed as the point‐of‐use devices to remove Hg(II) ions. The TLMSM exhibits remarkable adsorption performance with high selectivity (K_d_ > 5.0 × 10^7^ mL g^−1^), wide working pH range (1–10) and high capacity (954.7 mg g^−1^). DFT calculations and extended X‐ray absorption fine structure (EXAFS) have comprehensively confirmed the formation of different complexes based on hard–soft acid base theory in the adsorption process.^[^ ^139^ ^]^

In another typical research, Abney et al. transformed two indium MOFs (MIL‐68 or MIL‐53‐NH_2_) into four porous inorganic adsorbents by wet treatment method in Na_2_S solutions. These MOF template materials show obvious affinity for heavy metals, and can be saturated in less than 1 h. The EXAFS fitting results shows that each Hg connect with three S atoms, forming a robust cationic polymer, which is beneficial for its practical application (Figure 5d–g).^[^ ^140^ ^]^

### 3.3 Removal of Cu from Water

Copper itself is an essential trace mineral for human body and an effective catalyst for redox system. However, a large amount of copper will be ingested, which may lead to acute ketosis or chronic ketosis. Therefore, it is particularly important to treat copper in water. Next, we summarize several examples to remove copper with MOFs and MOF‐based adsorbents from water.

In a recent study, Wang et al. synthesized a new type of chitosan–MOF composites. At pH 5 and 60 °C, the adsorption capacity of Cu^2+^ was 50.6 mg g^−1^, and the pseudo‐second‐order adsorption mechanism was adopted.^[^ ^141^ ^]^ Similarly, Yu et al. have constructed a hydrogen‐bonded MOF [(Zn_3_L_3_(H_2_O)_6_][(Na)(NO_3_)] with high stability. This MOF can recognize and adsorb Cu ions through active sites according to the strong interaction between Cu ions and carboxylic acid oxygen atoms, and its adsorption capacity for copper ions is higher (379.13 mg g^−1^.^[^ ^142^ ^]^ The functionalized MIL‐101(Cr) with amino group (ED‐MIL‐101(Cr)) was synthesized. The maximum adsorption capacity of ED‐MIL‐101(Cr) for Cu^2+^ can reach 69.9 mg g^−1^. Amino‐functionalized MIL‐101(Cr) may be a potential candidate to remove metal ions from aqueous environment.^[^ ^143^ ^]^ Very recently, MOF‐199 coated with polyaniline (MOF‐199@PANI, core@shell) composite was prepared, which utilizes the porous structure of MOF‐199 and the complexation between Cu^2+^ and the N atom of imine in PANI. It can effectively and specifically adsorb Cu^2+^ in water, and the adsorption capacity reaches 7831.34 mg g^−1^, which is one to two orders of magnitude higher than other Cu^2+^ absorbents (including organic adsorbents or some carbon‐based). Moreover, the participation of PANI protect the MOF framework and also solves the shortcomings of common MOF, such as instability in water, low adsorption uptake, and poor selectivity.^[^ ^144^ ^]^

### 3.4 Removal of Cr from Water

In natural environment, chromium ions and the corresponding compounds are in the form of Cr^3+^ cations and oxyanions (HCrO_4_^−^, Cr_2_O_7_^2−^ or CrO_4_^2−^), which are carcinogens released from various industrial sewage. Note that hexavalent Cr(VI) is more toxic and mutagenic contaminant than Cr(III). Chromium ions can be lethal to living things in water and could cause nasal septum perforation, gastrointestinal diseases, leukopenia, and lung diseases. In light of that Cr(III) is low toxic and easily forms sediment such as Cr(OH)_3_, which can be readily removed. Below, we sum up several examples with MOF‐based materials to efficiently remove chromium ions.

In a typical work, Fang et al. used Na_2_CO_3_ mineralizer to prepare MIL‐100(Fe), which has excellent physical and chemical properties. With NaOH at 0.01 mol L^−1^, a high desorption efficiency of 73% was attained within 4 h, and the used MIL‐100(Fe)_Na_2_CO_3_ was desorbed and regenerated.^[^ ^145^ ^]^ Zhang et al. successfully prepared a new composite membrane (polyethylene imine (PEI)/MOF@polyvinylidene fluoride (PVDF)) by precipitating UiO‐66‐NH_2_ and then crosslinking it with PEI based on PVDF. Under the condition that the effective treatment capacity is 2322.23 L m^−2^ through the positive hole of electrostatic attraction belt, the waste concentration of Cr(VI) is broken through. PEI/MOF@PVDF has a high pure water flux of 561 L m^−2^ h^−1^, and the water flux of the membrane polluted by BSA can be restored to 96.13%. This membrane is easy to be reused and regenerated, and the newly developed composite membrane shows the potential to effectively remove Cr(VI) and Cr(III) in the treatment of raw wastewater.^[^ ^146^ ^]^

Furthermore, Valizadeh et al. prepared MOF@polymer beads, by embedded Zr‐BDC‐(NH_2_)_2_ into polymer beads utilizing polyethersulfone (PES), and designed an adsorption–photoreduction system to decrease the concentration of Cr(VI) below the drinkable level. This system can trap Cr(VI) decreasing concentrations to drinkable levels, release Cr(III), and reduce Cr(VI) to Cr(III) for the following adsorption/regeneration cycles.^[^ ^147^ ^]^ Very recently, Du et al. fabricated UiO‐66‐NH_2_(Zr/Hf) film membranes on an *α*‐Al substrate by reactive inoculation method. UiO‐66‐NH_2_(Zr/Hf) film showed superior photocatalytic Cr(VI) reduction performance under simulated and actual sunlight irradiation, and UiO‐66‐NH_2_(Zr) film could still maintain over 94% Cr(VI) reduction efficiency without degradation after 20 cycles. This MOF membrane photocatalyst provides a novel method for effectively photocatalytic elimination of pollutants in wastewater.^[^ ^148^ ^]^

### 3.5 Removal of U from Water

As radioactive nuclides, uranium element mainly exists in the form of UO_2_^2+^ in aqueous solutions, which is highly toxic and radioactive to the ecological environment. Separating and recovering wastewater solution and U(VI) from spent fuel can not only make good use of limited U(VI) resources, but also reduce the difficulty of subsequent treatment and environmental pollution.^[^ ^150^ ^]^ The removal of uranium is prominent for nuclear fuel production and human health.^[^ ^278^, ^279^, ^280^ ^]^ Currently, MOF‐based materials as adsorbents have gained terrific attention in the field of elimination of U(VI) from aqueous solutions.^[^ ^151^, ^152^ ^]^ Right after, some examples using MOF‐based materials for efficiently eliminate U(VI) in recent years are illustrated.

Experimentally, Hui et al. modified PCN‐222, which is highly durable and photoactive, with amino groups and phosphono, so that it can capture U^6+^ from aqueous solution. And under the irradiation of visible light, photoinduced electrons from the PCN‐222 host can effectively reduce U^6+^ pre‐enriched in the MOF structure, thus providing neutral uranium material, thus evacuating the PCN‐222 structure and easily regenerating active sites to capture more U^6+^. This automatic recovery process provides ultrahigh uranium extraction capacity that the number of adsorption sites does not limit. More importantly, compared with these nonredox competitive metal cations, the absorption selectivity of uranium is higher, and it can be used for uranium separation and pH range at a very wide uranium concentration, showing strong application potential.^[^ ^153^ ^]^ Zhang et al. grafted amino group to coordination unsaturated site by postsynthesis method, and prepared double‐shell hollow (DSHM) metal–organic skeleton functionalized by diaminomaleonitrile (DAMN) of chromium (III) p‐aminobenzoate. TEM, XRD, FT‐IR spectra, and nitrogen adsorption were used for describing the obtained DAMN‐functionalized hollow MOF. Amine‐grafted MIL‐101 has many active unsaturated metal sites and its hierarchical structure strengthened the contact between uranium and nitrogen atoms. The maximum adsorption capacity of uranium is 601 mg g^−1^. The optimal adsorption pH is 8, which is near to the pH of seawater. In addition, high adsorption rate and the high selectivity of uranium in simulated seawater provide a broad prospect for introducing DSHM–DAMN into seawater to extract uranium.^[^ ^154^ ^]^

**Figure 6:**
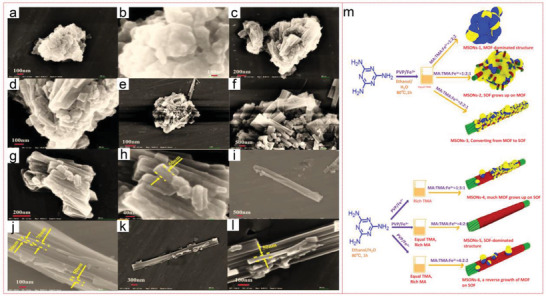
SEM images of the structural transformation process: a,b) MSONs‐1, c,d) MSONs‐2, e,f) MSONs‐3, g,h) MSONs‐4, i,j) MSONs‐5 and k,l) MSONs‐6, m) controlled‐switching of MSONs by the different molar ratios of MA, TMA, and Fe^3+^, and various morphologies associated with them. Reproduced with permission.^[^ ^149^ ^]^ Copyrigh 2020, Elsevier.

Furthermore, Li et al. also realized the synthesis of heterostructure framework (MSONs) between MOFs and supramolecular organic frameworks (SOFs) through self‐assembly method. MSONs adsorbent is directly formed by using MA containing N donor and TMA containing O donor as building units. Then, when they compete with MA to interact with the three O‐containing linkers on both sides of each TMA molecule, they insert high‐nuclear metal ions into the topological structure of SOF to capture a large number of active sites of architecture. By changing the topological structure from the original MOF‐based polyhedron to SOF‐based slender nanotubes, additional chemical modification steps and supramolecular agglomeration can be avoided, which allowing the controllable structure switching between MOF and SOF. Among six kinds of MSONs adsorbents, MSONs‐5 exhibited an extremely high UO_2_^2+^ loading capacity (526.6 mg g^−1^) during the morphological transformation process. Therefore, MA‐induced MSON can be regarded as a promising candidate for uranium sequestration (**Figure** **6**).^[^ ^149^ ^]^ The postsynthesis strategy of coordination, grafting coumarin on unsaturated Zn^2+^ center, can easily functionalize microporous–mesoporous Zn‐MOF‐74 to produce a various of coumarin‐modified Zn‐MOF‐74 materials. The obtained sample showed ultrahigh adsorption capacity for U^6+^ ions in water with pH value of 4, with the maximum adsorption capacity up to 360 mg g^−1^ (the recorded value of MOFs) and a remarkable optical switching capacity of 50 mg g^−1^ at pH value of 4.^[^ ^155^ ^]^

Accordingly, Alqadami et al. added 4.3 mmol FeCl_2_·4H_2_O and 8.7 mmol FeCl_3_·6H_2_O to the aqueous solution containing AMCA‐MIL‐53(Al) with different mass (0.00, 0.4 or 0.1 g) to synthesis Fe_3_O_4_@AMCA‐MIL53(Al). The suspension mixture was degassed and stirred and with nitrogen for 3 h, and then 20 mL of NH_3_ solution was added to obtain a black suspension. The magnetic metal–organic skeleton nanocomposite (Fe_3_O_4_@AMCA‐MIL53(Al)) was synthesized by filtering out the obtained material and washing it repeatedly with deionized water until the pH value became neutral. The adsorption of U(VI) reached equilibrium within 90 min. The adsorption capacity of U(VI) is calculated to be 227.3 mg g^−1^. Fe_3_O_4_@AMCA‐MIL‐53(Al) is a good material to remove these metal ions from aqueous media and the adsorbed metal can be easily recovered by desorption in 0.01 m HCl.^[^ ^156^ ^]^ Li et al. decorated ZIF‐8 nanoparticles on polypyrrole (PPy) nanotubes (PPy/ZIF‐8) in a simple and easy‐to‐operate way to obtain more MOF void spaces as active sites for capturing U^6+^. According to Langmuir model, the adsorption capacity at pH 3.5 is 534.0 mg g^−1^. The coordination of U^6+^ with pyrrole is the main reason for its excellent adsorption performance. At the same time, PPy/ZIF‐8 exhibited good selectivity and regeneration capacity, as well as good adsorption performance, which is a promising wastewater purification material.^[^ ^157^ ^]^ Acid‐resistant chromium‐based MIL‐101 and its amino derivatives were prepared. The adsorption capacity of these MOFs for U^6+^ follows the order of MIL‐101‐DETA > MIL‐101‐ED > MIL‐101‐NH_2_ > MIL‐101, among which MIL‐101‐DETA has the highest adsorption capacity: its capacity is 350 mg g^−1^ at pH value of 5.5. The adsorbed U^6+^ can be easily desorbed when the pH value is reduced (pH ≤ 3.0), and the material has ideal selectivity to U^6+^.^[^ ^158^ ^]^

**Figure 7:**
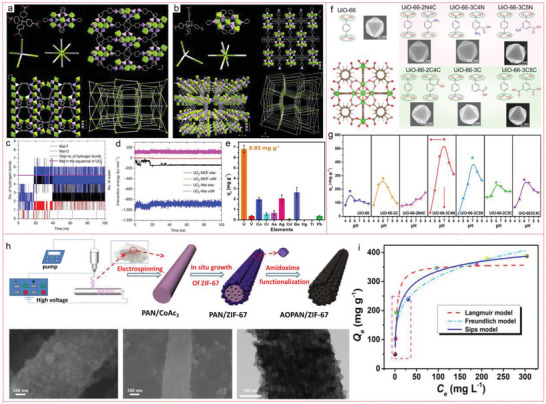
Network topology and crystal structure of a) SZ‐1 and b) SZ‐2. c) Time evolution of the electrostatic and vdW interaction energies of U(VI) with SZ‐2 and water. d) The number of hydrogen bonds formed between equatorial coordinating water molecules and other acceptors (including F and O in main framework) and the number of equatorial water molecules of U(VI) (pink curve) as the function of simulation time. e) U(VI) selectivity in natural seawater. Reproduced with permission.^[^ ^160^ ^]^ Copyright 2017, Springer Nature. f) Ligands composition and corresponding morphologies of UiO‐66s. g) Optimal pH for U(VI) capture. Reproduced with permission.^[^ ^161^ ^]^ Copyright 2022, Wiley‐VCH. h) The synthetic process of AOPAN/ZIF. i) Adsorption isotherms (C_0_ = 25–500 mg L^−1^, pH = 4). Reproduced with permission.^[^ ^162^ ^]^ Copyright 2020, Elsevier.

Very recently, Mei et al. also prepared high‐efficiency uranium adsorbent (ZIF‐90‐OM) with antibacterial property by synthesizing zeolite imidazole ester skeleton and functionalizing it with oxime. Due to the porous structure of ZIF‐90‐OM and the strong chelation of oxime groups with U^6+^, the maximum adsorption capacity for U^6+^ was 610 mg g^−1^ at pH = 5.0. Also, the adsorption capacity of the adsorbent remained unchanged after five adsorption/desorption cycles, which indicated its recyclability is good. ZIF‐90‐OM has antibacterial property and antifouling function, and can be used as adsorbent to purify water.^[^ ^159^ ^]^ Zheng et al. synthesized SZ‐2 and SZ‐3 by ionic thermal synthesis and hydrothermal reaction. Uranyl ions in aqueous solutions can be effectively removed by SZ‐2 and SZ‐3 over a wide pH range. SZ‐2 has the largest void volume recorded in zirconium phosphonate, and SZ‐3 represents the most porous crystalline zirconium phosphate, and is the only porous MOF material reported to survive in aqua regia (**Figure** **7**a–d).^[^ ^160^ ^]^ In another precedent, 4‐aminoisophthalic acid was introduced into UiO‐66 to prepare UiO‐66‐3CN. This modified MOF showed high uranyl adsorption capacity in natural seawater and seawater (Figure 7e–g), and showed high selectivity in natural seawater: the uranium extraction capacity of UiO‐66‐3CN was 17.03 times higher than that of vanadium, according to EXAFS analysis and DFT calculation.^[^ ^161^ ^]^ In addition, researchers in our group have prepared an organic–inorganic hybrid adsorbent by in situ anchored ZIF‐67 particles onto electrospinning polyacrylonitrile fiber (PAN), and then modified it with amidoxime groups to form amidoximed AOPAN/ZIF‐67 hybrid fiber. In this kind of fiber, the nitrogen atoms from imidazole and amidoxime can improve the synergistic adsorption performance in a wide pH range, which is beneficial to capture U(VI) wastewater and seawater under nuclear conditions. In addition, AOPAN/ZIF‐67 fiber showed a high adsorption capacity of 498.4 mg g^−1^ in the uranium contaminated aqueous solution with pH 4 (Figure 7h,i). This adsorbent behaved well in both wastewater (7.0 mg L^−1^) and artificial seawater (3.3 µg L^−1^), which was on account of the chelation between the imidazole‐N in ZIF‐67 and amidoxime.^[^ ^162^ ^]^

Besides, a new kind of carboxyl functionalized metal–organic skeleton have been prepared through a general postsynthesis strategy, in order to achieve high‐efficiency uranium adsorption with a saturated adsorption capacity of 314 mg g^−1^.^[^ ^163^ ^]^ De Decker et al. also selectively recovered uranium from water flow by adsorption of bottled CMPO in MIL‐101(Cr).^[^ ^164^ ^]^ Wu et al. utilized solvothermal method to synthesize rod‐shaped MOF‐5 nanomaterials, and applied it to effectively adsorb U(VI) from aqueous solution.^[^ ^165^ ^]^ The amidoxime‐functionalized porous material (MIL‐101‐AO), which was prepared by grafting amidoxime group onto chloromethylated MIL‐101(Cr), was used as an efficient adsorbent for recovering uranium from seawater.^[^ ^166^ ^]^ Zhang et al. have also developed a carbonized molecularly imprinted polymer (MIP)‐202/MXene composite material, which can be used to efficiently and selectively electroadsorb U(VI) from multi‐ion water.^[^ ^167^ ^]^ Feng et al. selectively recover uranium from seawater using uranium‐imprinted nanocages, MUU_im_, which derived from UiO‐66 via one‐step in‐situ synthesis approach.^[^ ^168^ ^]^

### 3.6 Removal of Others from Water

In addition to the above‐mentioned metal ions that pollute water bodies, there are other phenomena of water bodies pollution. Here we list several common water bodies pollution and give corresponding solutions.

#### 3.6.1 Oil–Water Separation

Many industries, such as textile, mining, food, petrochemical, and metal//steel industries, will produce a large amount of oily wastewater, which has become an exceedingly common pollutant all over the world and has now become a solemn global environmental problem. Therefore, it is very important to further develop cost‐effective, environment‐friendly, recyclable and reusable materials, and simple and effective oil–water separation technology that can purify a large amount of oil.^[^ ^169^ ^]^

**Figure 8:**
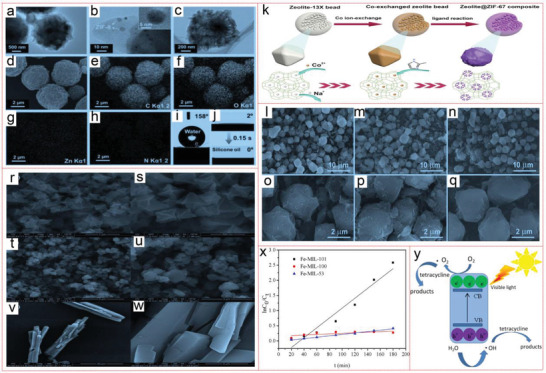
TEM images of a) overall view, b) local view with inserted high magnification, and c) plan view of the wrinkled ZIF‐8@rGO microsphere. d) SEM image and e–h) corresponding EDX mapping. Digital photo of i) water and j) silicone oil droplet profiles with contact angle values on the surface of the microsphere. Reproduced with permission.^[^ ^172^ ^]^ Copyright 2019, Wiley‐VCH. k) Schematic view of the synthetic process of zeolite@ZIF‐67 composites. SEM images of l,o) zeolite‐13X beads, m,p) coexchanged zeolite beads, and n,q) zeolite@ZIF‐67 composites. Reproduced with permission.^[^ ^176^ ^]^ Copyright 2022, Elsevier.SEM images of r,s) Fe‐MIL‐101, t,u) Fe‐MIL‐100, and v,w) Fe‐MIL‐53. x) Kinetics curve of pseudo‐first‐order equation for tetracycline degradation of Fe‐MILs. y) Schematic view for the separation and transfer of photogenerated electron–hole pairs in Fe‐MILs under visible light irradiation. Reproduced with permission.^[^ ^175^ ^]^ Copyright 2018, Elsevier.

Recently, highly fluorinated graphene oxide (HFGO) and nanocrystalline imidazole skeleton ZIF‐8 was combined to prepare superhydrophobic/superoleophilic composites. The microstructure of HFGO@ZIF‐8 composites has fluorine groups bonded on graphene. Self‐assembly of a unique micro–mesoporous structure was realized, in which the micropores originated from ZIF‐8 nanocrystals, while the functionalized mesopores came from randomly organized HFGO layers separated by ZIF‐8 nanopillars. The hybrid material shows extraordinarily high water contact angle of 162° and low oil contact angle of 0°, so it shows fast kinetics, very high adsorption selectivity and good absorption for nonpolar/polar organic solvents and oil in water. Therefore, Sponge@HFGO@ZIF‐8 composite has been successfully used for oil–water separation.^[^ ^170^ ^]^ Nowadays, oil leakage and oil‐polluted water need to be solved urgently, because it has become a worldwide problem. Therefore, it is imperative to synthesize functional materials for efficiently treating oily wastewater. Although many efforts have been made, the research in this field is still in the initial stage.^[^ ^171^ ^]^ By embedding MOF nanoparticles between graphene oxide (GO) nanosheets, and then self‐assembling at high temperature, Gu et al. developed a novel wrinkled 3D microsphere MOF@reduced GO (rGO) composite with superhydrophobic and superlipophilic properties. Microsphere composite composed of rGO nanosheets and well‐dispersed MOF nanoparticles has a one‐of‐a‐kind micro–nanostructure, which combines wettability with rich mesopores/micropores. The special structure of ZIF‐8@rGO microspheres shows fast absorption rate and high adsorption selectivity for oils and organic solvents in water (**Figure** **8**a–j).^[^ ^172^ ^]^ Li et al. successfully prepared high‐performance ZIF‐90 by adding triethylamine in 5–30 min at room temperature. On this basis, hydrophobic ZIF‐90‐CF_3_ ligand with low surface energy was designed. As the hydrophobic material ZIF‐90‐CF_3_ on the surface of melamine foam (MF) has porous structure, it not only has strong adsorption capacity for oil, Moreover, after oil absorption, an “oil film” can be formed, thus improving the hydrophobicity and lipophilicity of ZIF‐90‐CH3/MF, which has a good OC_S_‐water separation capacity, which can reach (40.1‐108.7 g g^−1^).^[^ ^173^ ^]^

#### 3.6.2 Tetracycline

Improper ingestion of tetracycline may cause adverse gastrointestinal reactions, such as nausea and vomiting, abdominal distension, diarrhea, hepatotoxicity, high liver function, tooth and bone development, rash, fever, asthma, photosensitivity dermatitis, and so on. Therefore, it is also very important to remove tetracycline from water.

In a recent study, a new type of core–shell In_2_S_3_@MIL‐125(Ti) (MLS) photocatalytic adsorbent was successfully prepared by simple solvothermal method. MLS shows excellent adsorption capacity for tetracycline (TC) in water through surface complexation, *π*–*π* interaction, hydrogen bond, and electrostatic interaction. In the TC degradation experiment in the presence of core–shell MLS under visible light, the optimum additive content of MIL‐125(Ti) in the synthesis process is 0.1 g, and the corresponding TC photodegradation efficiency is 63.3%. The core–shell MLS composite material also shows good performance in removing TC from actual wastewater (including medical wastewater, river water, and municipal wastewater). Therefore, the new hybrid can be taken as a promising photocatalytic adsorbent for wastewater purification.^[^ ^174^ ^]^ Wang et al. have also synthesized Fe‐MIL‐101, and its removal rate of tetracycline can reach 96.6% (initial tetracycline concentration is 50 mg L^−1^), while Fe‐MIL‐100 and Fe‐MIL‐53 remove 57.4% and 40.6% under the same conditions. And with the increase of time, the adsorption and photocatalytic degradation effect is better. The optimum addition amount of Fe‐MIL‐101 is 0.5 g L^−1^. With the increase of initial tetracycline concentration, the removal efficiency (RE) decreases. In addition, the trapping experiment and ESR test show that ·O_2_−, ·OH, and h^+^ are the main active substances in the photocatalytic degradation of tetracycline. Because of its simple synthesis and high removal efficiency, it can be used as a potential catalyst to degrade other antibiotics and tetracycline (Figure 8r–y).^[^ ^175^ ^]^ Chen et al. prepared ZIF‐67 supported on zeolite beads by organic ligand reaction and Co ion exchange. This method is easy, recyclable, and economical. The obtained zeolite@ZIF‐67 composite was used for PMS activation to degrade tetracycline in water. The uniform loading of ZIF‐67 enables 93.7% tetracycline (50 mg L^−1^) to be removed within 60 min by providing abundant catalytic sites, which has high recovery stability. Next, the zeolite@ZIF‐67 composite material in the form of microspheres can be readily filled into the bed column, thus effectively removing tetracycline in the continual flow process (Figure 8k–q).^[^ ^176^ ^]^

#### 3.6.3 Chemical Warfare Agents (CWAs)

In some parts of the world, wars still occur from time to time. With the progress of science and technology, the means used in wars have become more and more despicable. Some countries use chemical warfare agents in disregard of the regulations of the United Nations, causing large‐scale injuries or even deaths of people and livestock. These chemical warfare agents not only endanger human beings and livestock, but also use highly toxic synthetic organophosphorus, which can not only straightly destroy biological functions, but also cause secondary pollution of water and soil. Therefore, it is necessary to remove the residues of chemical warfare agents in water.

In the past, Zr‐based MOF can be regarded as a good degradation catalyst for CWAs in general, but its application is also limited due to its difficult‐to‐treat powder form and complicated instrument setup. In this case, Lee et al. developed a mixed matrix membrane reactor (MMR) based on Zr‐based MOF embedded in porous polymer matrix. This configuration also enables CWAs to flow through the nanochannels of MMR, thus facilitating contact with the catalyst and enhancing the degradation effect of CWAs. All kinds of flat MMR and hollow fiber MMR containing UiO‐66, UiO‐66‐NH_2_, and MOF‐808 were also evaluated. Despite the short residence time, MMR containing MOF‐808 still provided an extremely high transformation rate of 95% (**Tables** 1, **2**).^[^ ^177^ ^]^

**Table 2:** The adsorption capacity of different adsorbents for gases by MOF‐based materials

Target gas	Sample	Condition	BET surface area	Capacity	Refs.
CO_2_	WM‐MOF	*T* = 273 K	1724 m^2^ g^−1^	60 cm^3^ g^−1^	[226]
CO_2_	JUC‐1000	*T* = 273 K *P* = 0–1 bar	–	125 cm^3^ g^−1^	[224]
CO_2_	Mg‐CUK‐1	RH = 13% *P* = 2–6 bar	586 m^2^ g^−1^	6.39 mmol g^−1^	[227]
CO_2_	Zr‐UiO‐66‐SH‐h	*T* = 273 K	227.5 m^2^ g^−1^	2.88 mmol g^−1^	[228]
CO_2_	NJU‐Bai35	*T* = 298 K *P* = 0.15 bar	862.8 m^2^ g^−1^	7.20 wt%	[230]
SO_2_	DMOF‐M	*T* = 293 K *P* = 0.97 bar	1557 m^2^ g^−1^	12.15 mmol g^−1^	[7]
SO_2_	DMOF‐DM	*T* = 293 K *P* = 0.97 bar	1343 m^2^ g^−1^	10.40 mmol g^−1^	[7]
SO_2_	NU‐200	*T* = 298 K *P* = 1 bar	–	11.7 mmol g^−1^	[32]
SO_2_	MOF‐Fe‐soc‐MOF	*T* = 298 K *P* = 1 bar	1470 m^2^ g^−1^	11.7 mmol g^−1^	[234]
SO_2_	MOF‐177	*T* = 293 K *P* = 1 bar	4100 m^2^ g^−1^	25.7 mmol g^−1^	[236]
SO_2_	EDTA‐MOF‐808	*T* = 273 K *P* = 0.2 bar	1036 m^2^ g^−1^	9 mmol g^−1^	[265]
SO_2_	Cu_x_O@ASC‐3	*T* = 368 K	506.62 m^2^ g^−1^	233.11 mg g^−1^	[266]
SO_2_	DUT‐4	*T* = 298 K *P* = 1 bar	–	14 mmol g^−1^	[267]
SO_2_	Zr6‐NU‐907	*T* = 298 K *P* = 1 bar	–	4.9 mmol g^−1^	[268]
PM_2.5_	MiL‐53(Al)@Aramid	PM_2.5_ > 280 µg m^−3^	69.79 m^2^ g^−1^	RE = 95.30%	[239]
PM_10_	MiL‐53(Al)@Aramid	PM_10_ > 360 µg m^−3^	69.79 m^2^ g^−1^	RE = 96.11%	[239]
PM_0.3_	Cu//Tb SBS‐NFs	*T* = 77 K (PD = 60.7 ± 0.9 Pa	61.3 m^2^ g^−1^	RE = 90.2% ± 0.3%	[240]
PM_2.5_	Zr‐MOF‐NO_2_/cotton	*T* = 77 K PD = 31 Pa	–	RE = 89.5%	[241]
PM_10_	Zr‐MOF‐NO_2_/cotton	*T* = 77 K PD = 31 Pa	–	RE = 91.0%	[241]
PM_2.5_	PES@ZIF8‐PSA/PES	PD = 15 Pa	–	>78 µg m^−3^	[242]
PM_0.3_	PES@ZIF8‐PSA/PES	PD = 15 Pa	–	RE = 99.95%	[242]
PM_2.5_	Chitosan/PEO@MOF‐5 membrane	PD = 44 Pa *T* = room temperature Flow rate adopted = 3.4 m^3^ h^−1^	87.42 m^2^ g^−1^	FE = 99.95%	[243]
NH_3_	SION‐10	T = 303 K *P* = 1 bar *P*_NH3_:*P*_N2_ = 0.1 bar: 0.9 bar	–	27.3 mmol g^−1^	[248]
NH_3_	MFU‐4/fiber composite	*P* = 1 bar *T* = 298 K	–	17.7 mmol g^−1^	[250]
NH_3_	[BOHmin][Zn_2_Cl_5_]@MIL‐101Cr	*T* = 298 K *P* = 1 bar	2796.7 m^2^ g^−1^	24.12 mmol g^−1^	[251]
NH_3_	MOF‐303(Al)	*T* = 298 K *P* = 1 bar	1292 m^2^ g^−1^	19.7 mmol g^−1^	[253]
NH_3_	Fe‐soc‐MOF	*T* = 298 K *P* = 0.1 bar	1470 m^2^ g^−1^	6 mmol g^−1^	[269]
NH_3_	Zr6‐NU‐907	*T* = 298 K *P* = 1 bar	–	12.1 mmol g^−1^	[268]
CH_4_	MAX‐MIL composite	*T* = 160 K *P* = 10 bar	2670 m^2^ g^−1^	220 cm^3^ cm^−3^ 0.35 g g^−1^	[256]
CH_4_	HKUST‐1	*T* = 298 K *P* = 65 bar	1850 m^2^ g^−1^	267 cm^3^ cm^−3^	[259]
CH_4_	MAF‐38	*T* = 298 K *P* = 65 bar	2022 m^2^ g^−1^	263 cm^3^ cm^−3^	[270]
CH_4_	NU‐111	*T* = 298 K *P* = 65 bar	4930 m^2^ g^−1^	206 cm^3^ cm^−3^	[259]
CH_4_	NU‐125	*T* = 298 K *P* = 65 bar	3286 m^2^ g^−1^	232 cm^3^ cm^−3^	[259]
CH_4_	Al‐soc‐MOF‐1	*T* = 298 K *P* = 65 bar	5585 m^2^ g^−1^	197 cm^3^ cm^−3^	[271]
CH_4_	UTSA‐76	*T* = 298 K *P* = 65 bar	2820 m^2^ g^−1^	257 cm^3^ cm^−3^	[272]
NO_2_	UiO‐66‐NH_2_	RH = 80%	–	1.4 g g^−1^	[260]
NO_2_	MFM‐300(V)	*T* = 298 K *P* = 1 bar	–	13.0 mmol g^−1^	[261]
H_2_S	BiO(H_2_O)(C_14_H_2_O_8_)·NH_2_O(SU‐101)	*T* = 298 K *P* = 1 bar	412 m^2^ g^−1^	15.95 mmol g^−1^	[263]

## 4 Application of MOF in Air Environment

Air pollution will do harm to plants, making the surface of plant leaves damaged or withered off; acid rain will corrode and break paper products, textiles, and leather products; it will produce smog, affecting visibility; it will do harm to human lungs and cause respiratory diseases. Therefore, it is very important to control air pollution. MOF‐based material is often used to treat air pollution (Table 2)^[^ ^59^, ^207^, ^208^, ^209^, ^210^, ^211^, ^212^, ^213^, ^214^, ^215^, ^216^, ^217^ ^]^ because of its strong adsorption capacity^[^ ^213^, ^218^, ^219^, ^220^ ^]^ and catalytic performance.^[^ ^221^ ^]^

### 4.1 CO_2_

CO_2_ makes the greenhouse effect serious, leads to extreme weather such as typhoons and rainstorms, and also leads to the greenhouse effect, resulting in higher and higher global temperature and higher sea level. Thus, it is very important to deal with excess CO_2_ in the environment.^[^ ^222^, ^223^, ^224^ ^]^

**Figure 9:**
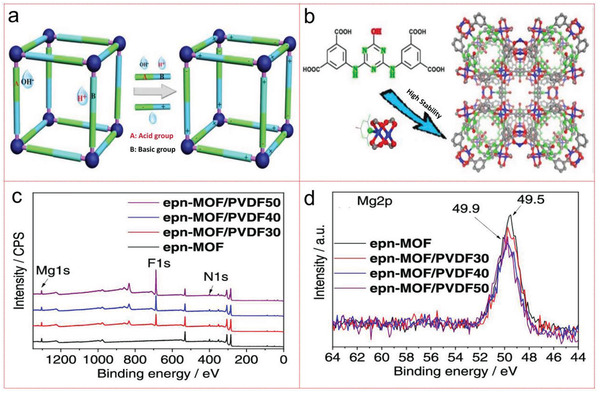
a) Schematic illustration of buffer strategy in JUC‐1000. b) Illustration of the buffer strategy for the construction of JUC‐1000. Reproduced with permission.^[^ ^224^ ^]^ Copyright 2018, Wiley‐VCH. c) XPS profiles of epn‐MOF, epn‐MOF/PVDFX (X = 30, 40, 50). d) High‐resolution spectra of Mg 2p electron peak for epn‐MOF/PVDF40. Reproduced with permission.^[^ ^229^ ^]^ Copyright 2021, American Chemical Society.

**Figure 10:**
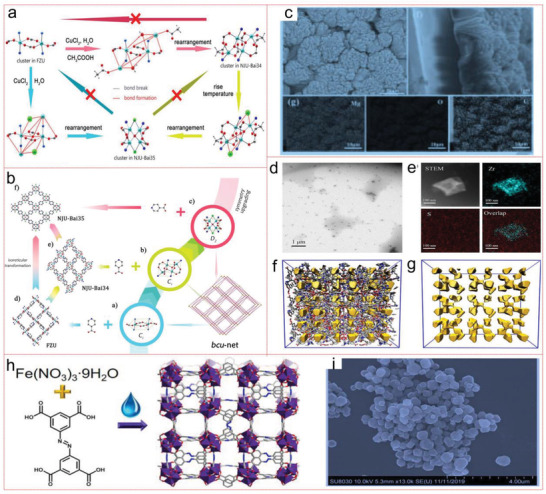
a) Main mechanism during the symmetry‐upgradingly isoreticular conversion. b) Isoreticular conversion of bcu‐type MOF by symmetry upgrading inorganic clusters. Reproduced with permission.^[^ ^230^ ^]^ Copyright 2018, American Chemical Society. c) Top view and cross‐sectional SEM images of the as‐grown Mg‐MOF‐74 film on IDEs. EDS elemental mapping images of the Mg‐MOF‐74 film. Reproduced with permission.^[^ ^231^ ^]^ Copyright 2019, Wiley‐VCH. d) TEM image and e) STEM‐EDS mapping of Im‐UiO‐PL. f) The snapshots of simulation boxes and g) pore space in Deim‐UiO‐66.^[^ ^233^ ^]^ h) Schematic representation of water‐based synthesis of Fe‐soc‐MOF. i) SEM image of Fe‐soc‐MOF. Reproduced with permission.^[^ ^234^ ^]^ Copyright 2020, American Chemical Society.

In a representative example, Chong et al. have obtained a sensing mode fiber for CO_2_ at the wavelength of 1.57 µm through a single layer of ultrasensitive NIR gas coated with nanoporous Cu‐BTC. This is the first time that we have obtained the high‐resolution NIR spectra of CO_2_ adsorbed in MOF, without any rotating sidebands, which indicates that the gas molecules enclosed in MOF pores do not have any rotational freedom. Most importantly, the single‐mode fiber with a 5 cm long Cu‐BTC film was coated, and the ultralow detection limit of CO_2_ (<20 ppm) was achieved.^[^ ^225^ ^]^ Furthermore, He et al. also have synthesized a series of zirconium‐metalloporphyrins MOF by introducing H_2_. And water monocarboxylic acid as regulators, organized mesoporous channels can be clearly observed under customary transmission electron microscope. Because a large number of unsaturated Lewis acid catalytic sites are revealed in the visualized mesoporous channels, these structures show excellent stability and excellent catalytic activity in chemically fixing CO_2_ into cyclic carbonate.^[^ ^226^ ^]^ Recently, He et al. designed and synthesized organic ligands with buffering effect, which greatly improved the water stability of porous MOF (JUC‐1000), and it kept its structural integrity at both high and low pH values. The local buffer environment caused by the weak acid–base pair of the customized organic ligand also greatly promoted the performance of JUC‐1000 in fixing CO_2_ chemically under environmental conditions, and its performance was superior to that of a series of benchmark catalysts (**Figure** **9**a,b).^[^ ^224^ ^]^ An eco‐friendly material Mg‐CUK‐1 was synthesized in water, which can adsorb acid gases such as CO_2_ with high capacity and high reversibility. Mg‐CUK‐1 was proved to maintain long‐term crystallinity after adsorption cycle. Even in the case of high relative humidity (95%), its adsorption performance can be maintained in multiple cycles. Mg‐CUK‐1 is used as an effective solid adsorbent in the field of acid gas capture, and its application is highly related to the purification of many industrial gas streams.^[^ ^227^ ^]^ Accordingly, Du et al. have changed hydrophilic Zr(Hf)‐UiO‐66 into superhydrophobic Zr(Hf)‐UiO‐66‐SH‐*y* (SH = thiol, *y* = fluoroalkyl) by introducing long‐chain fluorine substitution through the click reaction of organic linker mercaptan‐alkene. The water contact angles of four improved UiO‐66 are all greater than 150°. The grafted fluorine group with low surface energy becomes an effective protective layer of MOF, which makes it show splendid stability under extreme conditions such as alkalinity (pH = 12), high concentration NaCl solution (20 wt%), and saturated HCl. The Zr‐UiO‐66 MOFs grafted with 1*H*,1*H*,2*H*‐perfluoro‐1‐hexene have high CO_2_ adsorption contents of 1.54 and 2.88 mmol g^−1^ at 298 and 273 K, respectively.^[^ ^228^ ^]^ Park et al. have made millimeter MOF/PVDF composite beads with different amounts of PVDF binder (30, 40, and 50 wt%) by phase inversion and postfunctionalization of 1‐ethylpropane‐1,3‐diamine (epn). The composite can effectively degrade CO_2_ and it can be exposed to 60% Relative Humidity (RH) humidity at room temperature for up to one month. The CO_2_ absorption performance of MOF/PVDF40 with 40% PVDF loading is good, and it also shows excellent recyclability at low desorption temperature of 70 °C, which is good for saving energy for repeated use (Figure 9c,d).^[^ ^229^ ^]^ Jiang et al. have fine‐tuned the polynuclear cluster of MOF and bcu‐type MOF through the network transformation such as symmetry upgrade, and {[Cu_4_(µ_4_‐O)Cl_2_(IN)_4_][CuCl_2_]}_∞_ (NJU‐Bai_35_; NJU‐Bai for Nanjing University Bai group) synthesized the cluster with higher symmetry [Cu_4_(µ_4_‐O)Cl_2_(COO)_4_N_4_]. The symmetry triggered the adjustment of channels in NJU‐Bai_35_ to adapt to the principle of CO_2_ molecules, which makes the NJU‐Bai_35_ have high CO_2_ adsorption capacity (7.20 wt % at ≈0.15 bar and 298 K) (**Figure** **10**a,b).^[^ ^230^ ^]^ Besides, Yuan et al. also have grown homogeneous Mg‐MOF‐74 thin films in situ by appropriate metal ligand ratio. The postsynthesis improvement of ethylenediamine Mg‐MOF‐74 membrane can reduce the sensitivity to benzene (≈60%) by using the porosity reduction caused by amine coordination and the plugging of metal openings, and can improve the selectivity to CO_2_ (≈25%) by using the specific principle of interaction between amine and CO_2_. This study proves the practicability of adjusting the gas sensing characteristics by adjusting the interaction between Mg‐MOF‐74 and analyte, thus providing a new perspective for the development of sensor based on Mg‐MOF‐74 (Figure 10c).^[^ ^231^ ^]^ CO_2_ is easier to handle and store safely, compared with acetylene, because it has a wide tolerance.^[^ ^232^ ^]^ Zou et al. prepared MOF liquid (Im‐UiO‐PL) by surface ionization of imidazolium functional framework with steric inhibition (PEGS) crown. The adsorption capacity of Im‐UiO‐PL for CO_2_ can reach about 14 times that of pure PEGS. Unlike the solid counterpart of porous MOF, the stored CO_2_ can be released slowly in Im‐UiO‐PL and effectively used to synthesize cyclic carbonate in the atmosphere. This is the first example of using porous MOF liquid as catalytic CO_2_ storage material. It provides a new method for preparing unique porous liquid MOF with functional behaviors in diverse fields of catalysis and gas adsorption (Figure 10d–g).^[^ ^233^ ^]^

### 4.2 SO_2_

Sulfur dioxide does great harm to human body, and is easily absorbed by wet mucosal surface, producing sulfurous acid and sulfuric acid. Mild sulfur dioxide poisoning can cause tears, photophobia, sore throat, cough, and other symptoms. In severe poisoning, pulmonary edema will occur within a few hours. The chronic effect of sulfur dioxide is that systemic symptoms such as headache, dizziness, fatigue, and respiratory damage such as chronic rhinitis, pharyngitis, and bronchitis will occur in long‐term low‐density contact. Sulfur dioxide will form acid rain and fog, which will corrode buildings, forests lakes, groundwater, buildings, forests, ancient cultural relics and people's clothes. At the same time, long‐term acid rain will also cause vast losses to water and soil quality. Therefore, it is necessary to treat sulfur dioxide in the air.^[^ ^7^ ^]^

Gong et al. have prepared the best pore diameter DABCO by increasing the methyl density at the junction of benzene dicarboxylic acid ester in [Ni_2_(BDC‐X)_2_] (BDC‐X = mono‐, di‐, and tetramethyl‐1,4‐phthalate/terephthalate; DABCO = 1,4‐diazabicyclo [2,2,2] octane). Monte Carlo simulation and first‐principles DFT calculation prove that the methyl groups in the pore surface have strong affinity for SO_2_, which is due to the increase of steric hindrance and hydrophobicity caused by the increase of methyl density compared with the parent MOF. DMOF‐M and DMOF‐DM showed higher SO_2_ adsorption capacity (12.1 and 10.4 mmol g^−1^) under the pressure of 1 bar.^[^ ^7^ ^]^ Highly porous and strong cage NU‐200 with Cu_2_(CO_2_)_4_ as SBU was prepared by carefully assembling cyclohexane functionalized iron(II)‐clathrochelate‐based isophthalate joint. NU‐200 can adsorb and remove SO_2_ through the binding site in the best hydrophobic bag, which can reach 11.7 mmol g^−1^.^[^ ^32^ ^]^ Chen et al. prepared Fe‐based MOF‐Fe‐soc‐MOF (also known as PCN‐250‐Fe and Fe‐MIL‐127) by water‐based synthesis method. The MOF used its tunability and high porosity to capture SO_2_, and the adsorption rate reached 11.7 mmol g^−1^ at 1 bar and 298 K. In three consecutive SO_2_ adsorption–desorption experiments (Figure 10h,i).^[^ ^234^ ^]^ Lee et al. have prepared ZIF‐8 by electrospinning polylactic acid fiber with controlled surface pores by using water vapor‐induced phase separation and in situ uniform coating of ZIF‐8 crystal growth method. The charged ZIF‐8 net can capture SO_2_ by corona and potential capture under static and dynamic air flow conditions, and has proved its applicability as a dual‐function filter for particulate matter and gaseous matter.^[^ ^235^ ^]^ It was shown unprecedented affinity between SO_2_KAST‐7 (NBOFFIVE‐1‐Ni) and KAUST‐8 (AlFFIVE‐1‐Ni). The MOF‐coated quartz crystal microbalance transducer is used to develop a sensor which can detect low concentration SO_2_ in the range of 25 to 500 ppm.^[^ ^62^ ^]^ NH_2_‐MIL‐125(Ti), MOF‐177, and MIL‐160 were prepared under solvothermal conditions. MIL‐160 has excellent theoretical selectivity of ideal adsorption solution (293 K, 124–128 at 1 bar; 353 K, 79–95 at 1 bar), and the breakthrough performance, high onset time, and high stability under wet and dry SO_2_ exposure. The splendid sorption capability of MIL‐160 could be explained by DFT simulation calculations and matching heat of adsorption for the binding sites O_furan_···S_SO2_ and OH_Al‐chain_···O_SO2_ (both ≈40 kJ mol^−1^) and O_furan/carboxylate_···S_SO2_ (≈55–60 kJ mol^−1^).^[^ ^236^ ^]^ Fan et al. used propyl‐fused imidazolyl dicarboxylate ligand as template to prepare huge transition metal–uranyl [Co_24_U_6_] drum‐shaped nanocages, and based on this nanocages, Cage‐U‐Co‐MOF was prepared. This material is an efficient and selective adsorbent, which can remove trace SO_2_ (ppm level) from SO_2_/CO_2_ or SO_2_/CO_2_/N_2_ mixture in both dry and wet conditions.^[^ ^237^ ^]^

### 4.3 PM_2.5_

PM_2.5_ is a small component of the earth's atmospheric composition, but due to the development of daily industry, society, automobile exhaust, and other wastes, PM_2.5_ is increasing in the air. PM_2.5_ will enter the lungs of human body through breathing, which will easily lead to cough, expectoration, serious damage to lung cells, affect respiratory function, and cause lung cancer, asthma, lung sclerosis, chronic bronchitis, and other diseases. It may also cause heart disease. Therefore, it is very important to control PM_2.5_ in the air.

**Figure 11:**
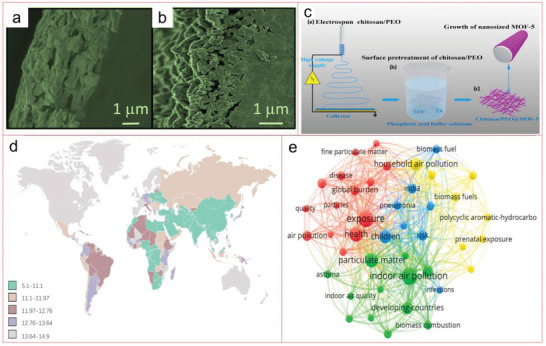
a) SEM of chitosan/PEO@MOF‐5 membrane, growth time of 30 s. b) Chitosan/PEO@MOF‐5 membrane, growth time of 2 min. c) Schematic illustration of electrospun chitosan/PEO, used as a skeleton to obtain a self‐supported and flexible chitosan/PEO@MOF‐5 membrane. Reproduced with permission.^[^ ^243^ ^]^ Copyright 2021, Elsevier. d) Score of Global Exposure to Air Pollution (Score: 0–100, 100 = Best, Time: 2019). e) Top keywords used about indoor air pollution. Reproduced with permission.^[^ ^245^ ^]^ Copyright 2021, Elsevier.

ZIF‐67 nanocrystals were integrated into electrospun PAN nanofibers using immersion method with excellent wind resistance and no membrane damage to prepare ZIF‐67@PAN filter. The filtration efficiency (FE) of this MOF composite for PM_2.5_ increased from 74.5% to 87.2%. In addition, after a 30‐day long‐term test, the filtration efficiency of PM_2.5_ remained above 99%. In addition, the removal efficiency of formaldehyde by ZIF‐67@PAN filter can reach 84%.^[^ ^238^ ^]^ Zhang et al. have prepared air filters by handling MOF onto textile substrates through environmentally amicable solvent‐free methods. The air filters synthesized by this method have microporous function, strong PM_2.5_ adhesion and other properties, and they are highly efficient and flexible. For example, when the average mass concentration of PM_2.5_ in MiL‐53(Al)@Aramid fiber is >280 µg m^−3^, the removal efficiency of PM_2.5_ is as high as 95.30%. These are promising composite materials, which can be used to produce efficient and recyclable outdoor/indoor air purifiers.^[^ ^239^ ^]^ In addition, Lee et al. have successfully manufactured the magneto luminescent Cu–Tb bimetallic MOF of side‐by‐side nanofiber (SBS‐NFs) films through self‐designed nozzle through electrospinning technology. Cu‐MOF is used to improve the leach efficiency and reduce the pressure drop (PD) by boosting the electrostatic interaction, while Tb‐MOF is used to study the adsorption process of PM through the change of luminous intensity. Sodium chloride (NaCl) aerosol with particle size not more than 300 nm was utilized for testing, and it is concluded that SBS‐NFs membrane doped with Cu//Tb MOF showed enhanced filtration efficiency (90.2%). Furthermore, the filter test with automobile exhaust shows that the PM removal efficiency of Cu//Tb SBS‐NFs membrane still keeps more than 91% within 30 h.^[^ ^240^ ^]^ Furthermore, Zr‐MOF, especially with functional groups, such as —NO_2_ was coated on cotton by covalent bonding to increase the removal efficiency of PM_2.5_. Even though the pressure drops of Zr‐MOF coating increases very little (less than or equal to 3 Pa), the quality factor (QF) and removal efficiency (RE) of Zr‐MOF‐NO_2_/cotton for PM_2.5_ removal are still 4.6 times and 6.2 times that of bare cotton, respectively. Here, the RE and QF of cotton with or without MOF was ranked: cotton < Zr‐MOF/cotton < Zr‐MOF‐NH_2_/cotton < Zr‐MOF‐NH‐SO_3_ H/cotton < Zr‐MOF‐NH_3_^+^Cl^−^/cotton < Zr‐MOF‐NO_2_/cotton. At the same time, it is confirmed that coating MOF on the substrate is a promising method to improve the PM removal performance of air filters, especially with large charge separation.^[^ ^241^ ^]^ Li et al. have prepared a unique layered composite nanofiber membrane PES@ZIF8‐PSA/PES by combining polyether sulfone (PES@ZIF8) fiber layer and polysulfonamide/polyether sulfone (PSA/PES) fiber layer embedded with zeolite imidazole ester skeleton‐8. With the advantages of multicomponent synergistic effect and double‐layered structure, the integrated PES@ZIF8‐PSA/PES filter uses its own multicomponent synergistic effect and double‐layered structure to achieve extremely low pressure drop (only 15 Pa), and super PM_0.3_ purification capacity (close to 99.95%) and long‐term recycling ability for purifying real smoke PM_2.5_ from >800 to <10 µg m^−3^. Besides, it has high temperature resistance (over 200 °C), flame retardancy, good chemical stability, satisfactory light transmittance, selective wettability, including hydrophobicity and super‐lipophilicity, and strong self‐cleaning ability. Especially PES@ZIF8‐PSA/PES nanofiber membrane can still maintain excellent oil/water separation and air filtration performance under strong acid/alkali conditions or high temperature. These comprehensive properties also make it have broad application prospects.^[^ ^242^ ^]^ Pan et al. used flexible and independent electrospun chitosan/(polyethylene oxide) (PEO) membrane fiber as the carrier of uniform and stable MOF‐5 to prepare chitosan/PEO@MOF‐5 nanofiber membrane. The MOF composite can provide a large number of cavities and gas adsorption sites through electrostatic interaction and polarization, and the filtration efficiency and quality factor of PM_2.5_ can reach 99.95% and 0.0737, respectively. Chitosan/PEO@MOF‐5 nanofiber membrane may be a great candidate for PM_2.5_ filtration (**Figure** **11**a–c).^[^ ^243^ ^]^ Very recently, Zhang et al. suspended the generated MOF particles in ultrapure water, and then deposited the MOF particles on two kinds of electret media with different lowest efficiency reported values (MERV 13 and 17) using a liquid filtration device to form an E‐MOFilter. Compared with cleaning electret media, E‐MOFilter only increases air resistance by a few Pa, but its PM removal efficiency is close to that of cleaning electret media. E‐MOFilter with MIL‐125‐NH_2_ particle coating not only has good toluene removal efficiency (>80%), but keeps its original PM_2.5_ retention capacity. This work may be helpful to apply the new E‐MOF filter to commercial and residential and HVAC systems and indoor air purifiers to remove PM_2.5_ effectively and VOC at the same time.^[^ ^244^ ^]^

Global air pollution is getting more and more serious. MOFs have a larger specific surface area than zeolite and conventional activated carbon, and have all kinds of skeleton structures, so it has super high adsorption capacity for PM_2.5_. But indoor air pollution is complicated and various. Achieving the goal of thoroughly improving indoor air quality by single treatment technology is difficult, so it is needed to manufacture composite materials. And we need to concentrate on reducing costs and improving scalability to produce these materials on an industrial scale (Figure 11d,e).^[^ ^245^ ^]^

### 4.4 NH_3_

NH_3_ is a colorless gas with strong pungent odor, which is harmful to human beings. Skin mucosa will become red, swollen, erythema, blisters, erosion, and even necrosis when exposed to ammonia, often accompanied by burning pain. When inhaling different concentrations of ammonia gas through respiratory tract, airway mucosa may be congested, edema, and secretion increase. In severe cases, pulmonary hemorrhage and chemical pneumonia may occur, and even sudden death may be caused by respiratory depression. Ammonia can also stimulate the digestive system, causing nausea, vomiting, and other uncomfortable symptoms; ammonia gas can also damage the nervous system. When the concentration of ammonia gas is too high, excessive inhalation can induce convulsions, convulsions, lethargy, coma, and other consciousness disorders, which can be life‐threatening. MOF is a promising candidate for capturing toxic gases, because their adsorption characteristics can be adjusted according to the topological structure and chemical composition of pores.^[^ ^246^ ^]^ Therefore, we introduce several schemes for treating ammonia in the environment.

**Figure 12:**
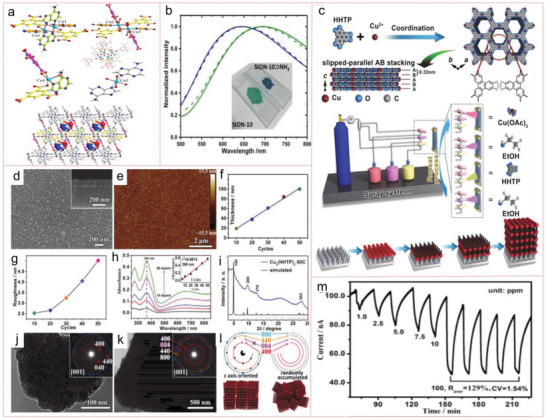
a) The structure of SION‐10. b) UV/vis spectra of SION‐10 (green) and SION‐10⊃NH_3_ (blue) and NH_3_ adsorption/desorption cycle. Inset: a digital photo showing the color difference after NH_3_ adsorption. Reproduced with permission.^[^ ^248^ ^]^ Copyright 2017, Wiley‐VCH. c) Schematic view of the crystal structure of Cu_3_(HHTP)_2_ and the assemble of its gas sensors. d) SEM image and e) AFM image of Cu_3_(HHTP)_2_‐40C. f) Thickness, g) roughness, and h) UV–vis spectra of Cu_3_(HHTP)_2_‐*x*C (inset: cycle dependent intensity of absorbance at 366 nm). i) PXRD patterns of as‐obtained Cu_3_(HHTP)_2_‐50C (blue) and simulation (black). TEM images and corresponding SAED patterns of fragments of j) Cu_3_(HHTP)_2_‐10C, k) Cu_3_(HHTP)_2_‐50C. l) Simulated SAED patterns of *c*‐axis oriented and randomly accumulated particles. m) The response–recovery curve toward NH_3_ with various concentrations. Reproduced with permission.^[^ ^249^ ^]^ Copyright 2017, Wiley‐VCH.

In the work of a representative, Zn(INA)_2_(H_2_O)_4_ (INA = isonicotinate) by interface reaction was employed to synthesize complex crystal films. The 3D Zn(INA)_2_ gained by dehydration of Zn(INA)_2_(H_2_O)_4_ can capture ammonia in several cycles under dry conditions without any influence on the structure. Furthermore, Zn(INA)_2_ can adsorb H_2_O_2_ and NH_3_ together in wet ammonia to shape a new material Zn(INA)_2_(H_2_O)_2_(NH_3_)_2_. Besides, the adsorption capacity of Zn(INA)_2_ to NH_3_ is 6 mmol g^−1^ under wet or dry conditions, and the adsorbent can be revived without loss of performance due to heating.^[^ ^247^ ^]^ Gladysiak et al. synthesized SION‐10 by mixing CuCO_3_ with 1,3,5‐trimellitic acid (H_3_btc) and adenine in pure water at 100 °C for 48 h. SION‐10 spontaneously absorbs NH_3_ due to chemical adsorption and physical adsorption (large permanent pores) caused by active copper. When NH_3_ was captured, the color of MOF changed from green to blue. When NH_3_ concentration was as low as 300 ppm, the change of UV/vis absorption band could be clearly seen. SION‐10 can be recovered upon immersion of SION‐10⊃NH_3_ in water and can be further recycled for NH_3_ capture for at least 3 cycles (**Figure** **12**a,b).^[^ ^248^ ^]^ Yao et al. have prepared controllable thin films of EC‐MOF, Cu_3_(HHTP)_2_, (HHTP = 2,3,6,7,10,11‐ hexahydroxytriphenyl) by layer‐by‐layer spraying liquid phase epitaxy. The chemical resistance gas sensor based on this high‐quality Cu_3_(HHTP)_2_ film is one of the best NH_3_ room temperature sensors among all reported sensors based on various materials (Figure 12c–m).^[^ ^249^ ^]^ Accordingly, Cao et al. also prepared MFU‐4 by using alcohol solvent at room temperature. Under this condition, MFU‐4 synthesized can be easily fixed on cotton textile fiber to capture NH_3_ well. The absorption of NH_3_ in MFU‐4/fiber composite reached the maximum (17.7 mmol g^−1^) at 1 bar, and MFU‐4 showed excellent NH_3_ capture performance of 10.8 mmol g^−1^ at low concentration (0.05 bar).^[^ ^250^ ^]^ [BOHmin][Zn_2_Cl_5_] was combined into the pores of MIL‐101(Cr) by slowly evaporating its methanol solution at room temperature, thus obtaining [BOHmim][Zn_2_Cl_5_]@MIL‐101(Cr). Due to the metal center of IL and hydroxyl functionalized alkyl chain, the MOF can capture NH_3_ by providing multiple adsorption sites and larger free volume, so as to achieve a higher NH_3_ capture (24.12 mmol g^−1^). In addition, it is found that the adsorbed water molecules in wet conditions can be used as additional adsorption sites to achieve excellent adsorption capacity for NH_3_. This work combines the advantages of IL and MOF, and provides a general method for developing adsorbents for capturing corrosive gases.^[^ ^251^ ^]^ Liu et al. use Zr_6_(µ_3_‐O)_4_(µ_3_‐OH)_4_(H_2_O)_8_^12+^ instead of Zr_6_(µ_3_‐O)_4_(µ_3_‐OH)_4_(H_2_O)_4_^8+^ and hydrogen bonding chloride ion on NU‐1000 to prepare NU‐1000‐Cl. NH_3_ was converted at the open Zr site into NH_4_^+^ by proton transfer from node aqua ligand and heat treatment. The existence of chloride ion seems to promote the irreversibility of the latter, because NH_4_^+^ is reversibly formed in chlorine‐free NU‐1000.^[^ ^252^ ^]^ Wang et al. use pyrazole‐3,5‐dicarboxylate with multiple sites as the best ligand to construct a solid MOF‐303(Al) with Al^3+^. At 25.0 °C and 1.0 bar, the adsorption capacity of MOF‐303(Al) to NH _3_ is as much as 19.7 mmol g^−1^ through hydrogen bonding interaction with NH_3_ sites, and the NH_3_ capture is completely reversible. No obvious loss of capture capacity was observed after 20 adsorption–desorption cycles.^[^ ^253^ ^]^

### 4.5 CH_4_

Although methane is fundamentally nontoxic to human body, when the concentration is too high, the oxygen content in the air is obviously reduced, which makes people stifle. When people are in the air with methane concentration of 25–30%, a series of clinical manifestations of hypoxia can appear, such as dizziness, headache, inattention, shortness of breath, weakness, ataxia, suffocation, etc. Methane can raise the global temperature, and excessive methane may also cause a global explosion. Therefore, it is necessary to treat methane in the environment. However, methane also has a good side. Methane is a very essential fuel and the main component of natural gas. It can be decomposed at high temperature to gain carbon black, which can be used as additives for pigments, paints, inks, and rubbers. Therefore, it is very meaningful for us to store methane.

**Figure 13:**
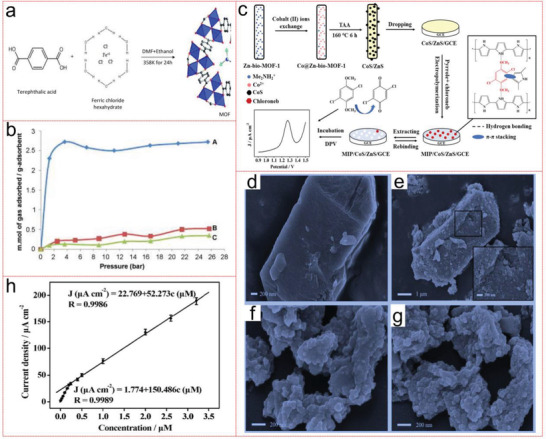
a) Synthesis of MOF‐235. b) Adsorption isotherms of CH_4_(A), H_2_(B), and CO_2_(C) on MOF‐235 at different pressure and 298 K. Reproduced with permission.^[^ ^257^ ^]^ Copyright 2012, Elsevier. c) Schematic synthetic process of MIP/CoS/ZnS/GCE and electrochemical detection of chloroneb. SEM images of d) ZnS, e) CoS/ZnS, f) MIP/CoS/ZnS/GCE before elution, and g) MIP/CoS/ZnS/GCE after elution. h) The calibration plots of current density versus chloroneb concentration and the peak potential at 1.272 V. Reproduced with permission.^[^ ^276^ ^]^ Copyright 2021, Springer Na.

Recently, Wang et al. dissolved Al(NO_3_)_3_·9H_2_O, H_4_PPTA, and propionic acid in DMF by ultrasound in a stainless steel autoclave lined with polytetrafluoroethylene, then heated in an oven at 150 °C for 48 h in an autoclave to obtain Al_3_(µ_3_‐O)(OH)(H_2_O)_2_(PPTTA)_3/2_ (BUT‐22). The weight storage capacity of BUT‐22 for CH_4_ can be as high as 530 cm^3^(STP) g^−1^.^[^ ^254^ ^]^ Kim et al. prepared UiO‐66‐Br_2_ by introducing various functional groups‐Br_2_ into the pores of hydrothermal stable zirconium‐based MOF. UiO‐66‐Br_2_ showed the highest selectivity of CH_4_/N_2_ under vacuum swing adsorption and pressure swing adsorption (PSA) (5.06 and 5.63 at 1 and 15 bar, respectively). Moreover, UiO‐66‐Br_2_ shows effective separation of CH_4_ from N_2_ under the condition of dynamic mixture flow, has good cyclic CH_4_ adsorption–desorption characteristics in 15 cycles, and is easy to regenerate under mild conditions without increasing the temperature.^[^ ^255^ ^]^ Besides, Kayal et al. prepared the proposed composite structure MAX‐MIL by in situ doping activated carbon powder (Maxsorb‐III type) into MIL‐101(Cr) by fluorine‐free hydrothermal reaction. XRD, SEM, TGA, and FTIR were used to measure the porosity, structure, morphology, thermal stability, and chemical function of MAX‐MIL composites. It was found that at 300 °C, the adsorption of CH_4_ by MAX‐MIL composite MOF was 9% (by volume) and 12% (by weight) higher than that of the parent MIL(Cr). Compared with the original MIL‐101(Cr), the composite MAX‐MIL adsorbed more CH_4_.^[^ ^256^ ^]^ Iron‐based MOF‐235 was synthesized by hydrothermal method, showing strong adsorption capacity for CH_4_ because of its high pore volume and a large number of open metal sites (**Figure** **13**a,b).^[^ ^257^ ^]^ Similarly, Tate et al. have prepared effective nanovalves by coating the MOF layer on zeolite 5A adsorbent. The obtained nanovalve material can seal the interior of high‐pressure CH_4_ adsorbent and store it at low pressure. Cheap, safe chemical, and renewable ethanol were utilized as nanovalve sealing molecule. The storage capacity of methane follows the following order: Al‐MOF > Ga‐MOF > Co‐MOF > Cu‐MOF. Among them, the material with the best performance (2‐crystallization step Al‐MOF) can store 33.6% (L (STP) CH_4_/L nanovalve adsorbent) of excess CH_4_, which is equivalent to the saturation of CH_4_ of zeolite 5A bead adsorbent when the pressure is less than 1 bar.^[^ ^258^ ^]^ Another example, HKUST‐1 was synthesized by using water/ethanol and a small amount of DMF. The total methane absorption of HKUST‐1 was about 230 cc(STP) cc^−1^ at 35 bar and 270 cc(STP) cc^−1^ at 65 bar. It was a commercially available material with gram level, and its room temperature methane absorption volume exceeded any value reported so far.^[^ ^259^ ^]^

### 4.6 Others

In addition to the above‐mentioned polluting gases in the air, there are many other polluting gases. Here we list several typical examples to introduce.

#### 4.6.1 NO_x_

Nitrogen oxides mainly damage the respiratory tract of human body. At the initial stage of inhalation, there may be only slight eye and respiratory tract irritation, and acute respiratory distress will occur several hours later. Therefore, it is necessary to treat nitrogen oxides in the air.

In a recent study, Peterson et al. have found that UiO‐66‐NH_2_ can remove nitrogen dioxide from the air by its own amine group, resulting in unprocessed removal capacities of 1.4 g g^−1^. This is for nitrogen dioxide, which leads to unprecedented removal capacity.^[^ ^260^ ^]^ Han et al. prepared redox active MFM‐300(V) to effectively adsorb NO_2_. The adsorption of NO_2_ leads to the oxidation of V(III) center to V(IV) center in MFM‐300(V), accompanied by the decrease of adsorbed NO_2_ to NO, and the release of water through deprotonation of skeleton hydroxyl groups, which is confirmed by various experimental techniques and synchrotron XRD. The effective accumulation of {NO_2_·N_2_O_4_}_∞_ chain in the pores of MFM‐300(VIV) resulted in the absorption of NO_2_ at high temperature of 13.0 mmol g^−1^ at 298 K and 1.0 bar, and kept several adsorption–desorption cycles.^[^ ^261^ ^]^

#### 4.6.2 H_2_S

H_2_S is harmful to human body, and can lead to mucous membrane irritation symptoms: eye stinging, burning, fear of light, blurred vision, tears, mucous membrane congestion, and severe keratitis. Respiratory tract damage: cough, itchy throat, chest pressure, even dyspnea, and acute pulmonary edema. It can also lead to hypoxia of nervous system: the patient can show dizziness, headache, lethargy, weakness of limbs, irritability, delirium, convulsion, and coma. When the inhalation concentration is too high, the patient may faint on the spot and die immediately. Therefore, it is very important to treat H_2_S in the environment.

Experimentally, Smith et al. have integrated conductive 2D MOF into fabrics by direct solution‐phase self‐assembly of simple molecular building blocks to prepare multifunctional electronic textiles. Self‐organizing framework equipment on textiles can detect and distinguish important gaseous analytes (NO, H_2_S, and H_2_O) in ppm level, and keep its chemical resistance function in the presence of humidity (5000 mg L^−1^, 18% RH). With theoretical detection limit of sub‐ppm (H_2_S = 0.23 mg L^−1^).^[^ ^262^ ^]^ Grape et al. prepared Bi_2_O(H_2_O)_2_(C_14_H_2_O_8_)·*n*H_2_O (SU‐101) by using reagent grade and affordable dietary supplement grade ellagic acid from bark and pomegranate shell. Its total absorption of H_2_S can reach 15.95 mmol g^−1^, which represents one of the highest H_2_S capacities, and polysulfide is formed in the pores of the material. Phenolic chemicals as the linker have opened up a new way to design stable, environmentally scalable, friendly, and low‐cost MOF for various applications, including drug delivery.^[^ ^263^ ^]^

#### 4.6.3 Cl_2_

Cl_2_ is a poisonous gas with strong asphyxiating odor. If chlorine gas is inhaled in high concentration for a long time, it may harm human skin mucosa, respiratory system, and gastrointestinal system. The mechanism of chlorine gas harm lies in its contact with mucous membrane and water to form harmful substances, thus playing a toxic role. Therefore, it is necessary to treat chlorine in the environment.

Song et al. have prepared nanopacked beds by synthesizing UiO‐66‐NH_2_ nanoparticles in the pores of microporous expanded polytetrafluoroethylene (ePTFE) membrane. The sub‐micrometer size of the membrane can enlarge the surface area of MOF nanoparticles, which can effectively capture/adsorb toxic gas molecules and react with them. The ePTFE membrane equipped with MOF can provide significant protection against the intrusion of Cl_2_ and 2‐chloroethyl ethyl sulfur (the simulant of sulfur mustard seed). The microporous membrane filled with reactive MOF nanoparticles can be used as a protective barrier against toxic gases/vapors (such as NH_3_ and Cl_2_), and still can basically permeate H_2_O and air.^[^ ^264^ ^]^

## 5 Application of MOF in Soil Environment

Soil pollution is due to the rapid development of modern industry. All kinds of industrial wastewater, harmful gases, and other pollutants enter the soil and accumulate to a certain amount. Cause quantitative change and qualitative change, and lead to soil destruction. There are many hazards of soil pollution, which will directly destroy the quality of crops, pollute groundwater and surface water, endanger human health, and affect the quality of atmospheric environment. Therefore, it is necessary to treat the pollutants in the soil.

Typically, Guselnikova et al. realized the surface‐assisted growth of MOF‐5 film by covalent grafting of 4‐carboxyphenyl, and then dipped the sample into the mother solution of MOF‐5. By functionalizing the surface of gold grating supported by surface plasmon polaritons with MOF‐5 for sensitive, selective and repeatable surface‐enhanced Raman scattering (SERS) is used to detect organophosphorus pesticides. The method has high reliability and low detection limit (up to 10–12 m). The uniform dispersion of plasma intensity along the surface of the gold grating ensures the high repeatability of SERS results (the deviation of Raman peak intensity along the sample is less than 4%).^[^ ^273^ ^]^ Li et al. synthesized MOF‐1210(Zr/Cu) with ordered spatial structure, large specific surface area, and permanent high porosity step by step by solvothermal method. Combined with high performance liquid chromatography, MOF‐1210(Zr/Cu) extracted benzophenones (BPs) by hydrogen bonding and coordination with BPs. The method has been successfully applied to the extraction and detection of benzophenone in soil samples. The recovery rate is 87.6–113.8%, and the relative standard deviation is less than 11.12%.^[^ ^274^ ^]^ Accordingly, Miliutina et al. form the plasma absorption band by depositing a thin gold layer on the bare core of multimode fiber. MOF‐5 layer was deposited on the surface of gold layer to achieve high affinity for target pesticides. MOF‐5 layer mainly provides the extraction and concentration of pesticides in the space of “plasma van evanescent wave,” thus allowing the detection through the movement of plasma absorption band. The synthesized sensor can be used to detect pesticides in soil without generating false signals from surrounding media. It has the advantages of simplicity, high sensitivity, low cost, and no organic solvent for probe treatment.^[^ ^275^ ^]^ Duan et al. used cobalt ion‐exchanged zinc‐based biological Zn‐bio‐MOF‐1 as precursor to synthesize CoS nanoparticles‐connected ZnS rods (CoS/ZnS composites) by solvothermal method. A novel electrochemical sensor based on CoS/ZnS complex and MIP is proposed, which can be used for sensitive rapid and highly selective detection of organochlorine pesticide chlorobenzene. Differential pulse voltammetry was employed to detect chloroneb. Under the optimum conditions, the oxidation peak current density of chlordecone is linearly connected with its concentration, with a detection limit of 0.87 nm (S/N = 3) and a sensitivity of 52.27 µA·µm ^−1^ cm^−2^ (Figure 13c–h).^[^ ^276^ ^]^

## 6 Conclusions and Outlooks

In this review, we have summarized the synthetic strategies of MOFs and MOF‐based composites, which offered great promise as adsorbents in environmental application, especially for water treatment and air purification. In recent years, large quantity of articles has reported the capture of toxic metal ions from aqueous solutions and the storage of hazardous gas from air atmosphere under the support of MOFs, exhibiting the significance of the related materials in this domain. In most of the cases, MOFs possess large specific surface area (1000–10 000 m^2^ g^−1^) and high porosities. Besides, the adsorption rate and removal efficiency can be enhanced by functionalizing or producing MOF‐based composites. To draw a conclusion, for MOFs and MOF‐based composites the impressive adsorption performance is mainly due to the affinity of the functional moiety on the MOFs and the target adsorbate, accompanying with the highly ordered and flexible porous structure, which can be rationally controlled to promote the diffusion of adsorbate. Thereby, when designing adsorbents, all the parameters (e.g., adsorption capacity, kinetic rate, selectivity, cyclability, and structural stability) should be taken into account to acquire optimal adsorption performance. Notwithstanding remarkable achievements in recent years, the unsolved issues and challenges also remain, among which their poor structural stability (under acidic, neutral and basic conditions) and low yield are extremely restricted the practical applications.

**Scheme 2:**
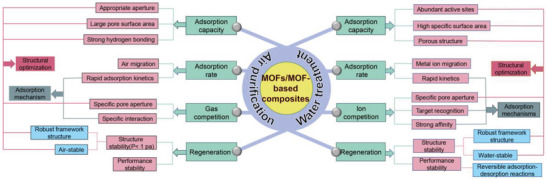
A summary of four aspects considered to design MOFs and MOF‐based composites for high performance adsorbents.

For water decontamination, i) generally, the solution conditions, especially pH and the coexistence of competitive adsorbate have substantial influence on the adsorption behavior of MOFs and MOF‐based composites; ii) the active pH range of certain MOFs is relatively narrow, thereby the performance stability of more MOFs or composites under a wider pH range is desperately needed in future study; iii) for practical applications, more structural‐stable MOFs or composites (under acidic, neutral, and basic aqueous environment) should be inquired; iv) to avoid secondary pollution, the regeneration and long‐term stability of MOFs and MOF‐composites should be improved for future research; v) simultaneous capture of multiple competitive ions toward MOFs and MOF composites is of great significance and needs to be further investigated for practical applications; vi) the catalytic conversion of some soluble metal ions into insoluble species is efficient to removal and enrichment of toxic metal ions (**Scheme** **2**).

For air purification, i) the tailorability of MOFs facilitates the modification of the organic linkers and SBU to target interactions with diverse toxic gases; ii) the structural stability and regeneration property are still challenging under relative pressure, which is rarely discussed in most reported works; iii) the hydrophilic nature and highly active sites offer low‐energy approach for the efficient removal of hazardous gases.

In the last decade, MOFs have come forth as an extraordinary material for manifold applications, and an increasing number of publications disclose that MOFs and MOF‐based composites can approach and exceed conventional materials in water treatment and air purification. Accordingly, more thorough theoretical investigations about adsorption behaviors and mechanisms are highly required. For practical applications, the high adsorption capacity, rapid kinetic rate, excellent selectivity, superior regeneration and reusability, and large‐scale production should also be taken into consideration in environmental governance.

## Conflict of Interest

The authors declare no conflict of interest.
